# Supplementary material for: Effects of X-ray–based diagnosis and explanation of knee osteoarthritis on patient beliefs about osteoarthritis management: A randomised clinical trial
Source: PLoS Med. 2025 Feb 4;22(2):e1004537. doi: 10.1371/journal.pmed.1004537 (PMC11838874; doi:10.1371/journal.pmed.1004537)
Supplement: S1 Appendix — (DOCX) [file pmed.1004537.s001.docx]

# S1 Appendix. Trial protocol

**Effects of a radiographic diagnosis and explanation of knee osteoarthritis on consumer beliefs about knee pain management: an online randomised controlled trial.**

|  |
| --- |
| **Authors**  Dr Belinda Lawford  Prof Rana Hinman  Prof Kim Bennell  Dr Dan Ewald  Ms Barbara Capewell  Dr Michelle Hall  Mr Travis Haber  Dr Thorlene Egerton  A/Prof Stephanie Filbay  A/Prof Fiona Dobson  Mr Jesse Pardo  Ms Peixuan Li  Dr Anurika De Silva  **Sponsor**  University of Melbourne |

**CONFIDENTIAL**

This document is confidential and the property of the University of Melbourne.

No part of it may be transmitted, reproduced, published, or used without prior written authorisation from the institution.

STATEMENT OF COMPLIANCE

This clinical trial will be conducted in compliance with all stipulation of this protocol, the conditions of the ethics committee approval, the NHMRC National Statement on ethical Conduct in Human Research (2007 and all updates), the Integrated Addendum to ICH E6 (R1): Guideline for Good Clinical Practice E6 (R2), dated 9 November 2016 annotated with TGA comments.

Last Modified:

22/03/2024

**PROTOCOL SYNOPSIS**

| Title | Effects of a radiographic diagnosis and explanation of knee osteoarthritis on consumer beliefs about knee pain management: an online randomised controlled trial |
| --- | --- |
| Objectives | 1. To evaluate the effects of a hypothetical diagnosis and explanation of knee OA based on x-rays (and showing a patient their x-ray images) on beliefs about knee pain management, compared to a diagnosis and explanation that does not involve using x-rays. 2. To evaluate the effects of a hypothetical diagnosis and explanation of knee OA based on x-ray (and showing a patient their x-ray images) on beliefs about knee pain management, compared to a diagnosis and explanation based on x-ray (and not showing the patient their x-ray images). |
| Study Design | A three-arm online superiority randomised controlled trial (RCT). |
| Planned Sample Size | 609 participants |
| Selection Criteria | We will recruit a sample of 50% who have knee pain, and 50% who do not have knee pain. Inclusion criteria are:  - adults aged 45 years or over  - currently living in Australia  - either have, or have not, experienced activity-related knee joint pain over the last 3 months   - have never consulted a healthcare professional for chronic knee pain, and;   - are able to easily understand written English |
| Study Procedures | The entire trial will be administered in one single online survey. Participants will initially complete screening questions to determine eligibility. Eligible participants will then be asked to imagine a hypothetical scenario where they have made an appointment with a GP to find out what is wrong with their painful knee. Participants will then be automatically randomised by Qualtrics to one of three groups, all of whom will be asked to watch a pre-recorded video on their screen where they receive a hypothetical knee OA diagnosis from the GP:   1. *Clinical diagnosis and explanation without being sent for x-ray*: the video will show the GP providing a clinical diagnosis of knee OA based on their age and symptoms, including an explanation of why x-rays are not necessary for OA diagnosis. The GP will then explain what OA is. 2. *Diagnosis and explanation based on x-ray findings (but without showing the x-ray images)*: the video will show the GP ordering an x-ray for the participant, summarising the x-ray report (but not showing the participant the x-ray images), and providing a diagnosis of knee OA. The GP will then explain what OA is. 3. *Diagnosis and explanation based on* x-ray (*and* *showing the x-ray images*): the video will show the GP ordering an x-ray for the participant, then summarising the x-ray report and showing them the x-ray images, and providing a diagnosis of knee OA. The GP will then explain what OA is.   All participants will complete outcome measures immediately after watching their allocated video. |
| Statistical Procedures  Sample Size  Analysis Plan | **Sample Size Calculation:**  The sample size is based on detecting a between-group difference of 1 NRS unit for both primary outcomes, beliefs about joint replacement surgery and beliefs about exercise and physical activity (based on data from a previous trial [3]). Assuming a between-participant standard deviation of 3 NRS units for both outcomes [3], to achieve 80% power, with a two-sided significance level of 0.0125 (conservative Bonferroni correction for two primary outcomes and two primary pair-wise comparisons), and accounting for no attrition [1], we require 203 participants per arm, or a total of 609 participants.  **Analysis Plan:**  A statistical analysis plan will be finalised prior to data analysis and published on our centre’s website. Statistical analyses will be performed blinded to group details. Similar to our prior online trials [1-3], primary and secondary outcomes post-intervention will be analysed using linear regression models. Results will be presented as estimated mean differences between groups, with two-sided 95% confidence intervals and p-values (multiplicity adjusted for primary outcomes). |
| Duration of the study | Data collection is anticipated to take one month (April 2024). |

GLOSSARY OF ABBREVIATIONS

| **Abbreviation** | **Term** |
| --- | --- |
| CTR | Clinical Trials Registry |
| BMI | Body Mass Index |
| CHESM | Centre for Health, Exercise and Sports Medicine |
| CONSORT | Consolidated Standards of Reporting Trials |
| CI | Confidence Interval |
| HREC | Human Research Ethics Committee |
| OA | Osteoarthritis |
| PLS | Plain Language Statement |
| RCT | Randomised Controlled Trial |
| SD | Standard Deviation |

Study management

Investigators

| **Name** | **Role** | **Contact information** |
| --- | --- | --- |
| Dr Belinda Lawford | Dr Lawford is a health scientist with a special interest in telehealth, exercise, and weight loss. She will lead the RCT, manage the budget, and coordinate the trial. She will be responsible for publishing the findings of the trial. | P: 03 8344 2045  E: [belinda.lawford@unimelb.edu.au](mailto:belinda.lawford@unimelb.edu.au)  Centre for Health, Exercise and Sports Medicine  Department of Physiotherapy  School of Health Sciences  University of Melbourne VIC 3010 |
| Prof Rana Hinman | Prof Hinman is a research physiotherapist who has conducted >30 RCTs in musculoskeletal conditions, most in knee OA. She will co-supervise the trial coordinator. | P: 03 8344 3223  E: [ranash@unimelb.edu.au](mailto:ranash@unimelb.edu.au)  Centre for Health Exercise and Sports Medicine  Department of Physiotherapy  School of Health Sciences  University of Melbourne VIC 3010 |
| Prof Kim Bennell | Prof Bennell is a research physiotherapist and Director of the Centre for Health, Exercise and Sports Medicine (CHESM). She will co-supervise the trial coordinator. | P: 03 8344 4135  E: [k.bennell@unimelb.edu.au](mailto:k.bennell@unimelb.edu.au)  Centre for Health Exercise and Sports Medicine  Department of Physiotherapy  School of Health Sciences  University of Melbourne VIC 3010 |
| Dr Dan Ewald | Dr Ewald is a General Practitioner and Public Health Physician, and  Adjunct Ass/Prof Sydney University Medical School. Northern Rivers University Centre for Rural Health. | GP Lennox Head Medical Centre. Ph 02 66877444  GP Bullinah Aboriginal Health Service. Ph 02 66815644 |
| Ms Barbara Capewell | Ms Capewell is a consumer with knee OA who will provide feedback on the study design and intervention. | P: n/a  Email: [zazyb1014@gmail.com](mailto:zazyb1014@gmail.com) |
| A/Prof Michelle Hall | A/Prof Hall is an exercise scientist with a special interest in the use of non-pharmacological treatments including exercise and weight loss for people with OA. She will assist the principal investigator to conduct the trial. | P: 03 8344 0556  E: michelle.hall@sydney.edyu.au  Sydney Musculoskeletal Health  School of Health Sciences  University of Sydney NSW 2050 |
| Mr Travis Haber | Mr Haber is a PhD candidate with expertise in OA education and diagnosis language. | P: N/A  E: [thaber@student.unimelb.edu.au](mailto:thaber@student.unimelb.edu.au) |
| A/Prof Thorlene Egerton | A/Prof Egerton is a research physiotherapist with expertise in GP management and education of people with lower limb OA. She will assist the principal investigator to conduct the trial. | Centre for Health, Exercise and Sports Medicine  Department of Physiotherapy  School of Health Sciences  University of Melbourne VIC 3010 |
| A/Prof Stephanie Filbay | DA/Prof Filbay is a research physiotherapist with a special interest in the use of non-pharmacological treatments including exercise for people with OA. She will assist the principal investigator to conduct the trial. | Centre for Health, Exercise and Sports Medicine  Department of Physiotherapy  School of Health Sciences  University of Melbourne VIC 3010 |
| A/Prof Fiona Dobson | A/Prof Dobson is a research physiotherapist with an interest in the use of non-pharmacological treatments including exercise for people with OA. She will assist the principal investigator to conduct the trial. | Department of Physiotherapy  School of Health Sciences  University of Melbourne VIC 3010 |
| Jesse Pardo | Mr Pardo is a research scientist with experience coordinating clinical trials of OA management. | Centre for Health, Exercise and Sports Medicine  Department of Physiotherapy  School of Health Sciences  University of Melbourne VIC 3010 |

Statisticians

| Fiona McManus | P: 03 8344 8701  E: [fmcmanus@unimelb.edu.au](mailto:fmcmanus@unimelb.edu.au)  Centre for Epidemiology and Biostatistics  Melbourne School of Population and Global Health  University of Melbourne VIC 3010 |
| --- | --- |
| Peixuan Li | P: 03 834 47697  E: [li.p4@unimelb.edu.au](mailto:li.p4@unimelb.edu.au)  Methods and Implementation Support for Clinical and Health research (MISCH) Hub  Faculty of Medicine, Dentistry and Health Sciences  University of Melbourne VIC 3010 |
| Dr Anurika De Silva | P: 03 8344 7846  E: anurika.de@unimelb.edu.au  Methods and Implementation Support for Clinical and Health research (MISCH) Hub  Faculty of Medicine, Dentistry and Health Sciences  University of Melbourne VIC 3010 |

Sponsor

The University of Melbourne.

Funding

National Health and Medical Research Council (NHMRC) Grant (#2025733).

INTRODUCTION AND BACKGROUND

Background Information

Knee osteoarthritis (OA) is a major public health problem and a leading cause of physical disability worldwide [4]. As there is no cure, people with OA are encouraged to engage in long-term self-management of symptoms, including exercising and losing weight (if necessary) as first-line approaches recommended in clinical practice guidelines [5-8]. However, uptake of exercise and weight loss in OA is low, and use of joint replacement surgery – recommended to be reserved for end-stage disease, or people who have not achieved adequate symptom management with conservative management approaches – is inappropriately high [9, 10].

One potential driver of inadequate uptake of conservative management approaches, and inappropriate use of joint replacement surgery, is inaccurate beliefs and misconceptions of OA among consumers. Given that OA is characterised by pain and physical dysfunction [11, 12], symptoms are often assumed to stem from degenerative structural changes within the joint [13]. However, research shows that pathoanatomical changes to the joint are poorly correlated with symptoms of pain and physical dysfunction, and do not necessarily predict prognosis of symptoms over time [12, 14, 15].

Radiographic imaging is not routinely recommended for determining diagnosis or prognosis of OA [12, 15], and a clinical diagnosis of OA can be made based on age and symptoms [8]. Despite this, recent research shows that many primary healthcare providers in Australia and overseas [10, 16] are still using imaging to diagnose OA in their patients. Further, people with OA expect imaging [17, 18]. There is evidence from qualitative studies that people with OA who see x-rays of their joint then believe that they need to protect their joint from further damage [17, 19]. However, to date, no empirical evidence from high-quality randomised controlled trials (RCTs) exists to support the premise that use of x-rays for diagnosis adversely impacts people’s beliefs about OA management. Outside of OA, an RCT in low back pain found that receiving ‘best practice’ care (without use of imaging) during diagnosis leads to more favourable beliefs about back pain severity and recovery, better satisfaction, and lower fear of movement compared to receiving imaging [20]. However, to our knowledge, no such trials have been conducted in a population with OA.

The aim of this study is to evaluate the effects of a clinical OA diagnosis and explanation without being sent for x-ray on consumer beliefs about OA management, compared to diagnosis and explanation based on x-ray reports (with and without x-ray images).

Research Questions

1) Does a hypothetical knee OA diagnosis and explanation based on x-rays (and showing the patient their x-ray images) impact beliefs about need for surgery and helpfulness of exercise, compared to a diagnosis and explanation that does not involve sending the patient for x-ray?

2) When explaining a hypothetical x-ray report of knee OA to a person, does showing the person the images of their x-ray lead to less favourable beliefs about need for surgery and helpfulness of exercise, compared to not showing them their x-ray images?

Rationale for Current Study

Management of people with knee OA is inconsistent with recommendations from evidence-based guidelines. Rates of joint replacement surgery and use of medications are inappropriately high [9, 10], and recommended first-line lifestyle approaches such as exercise and weight loss are under-utilised [9, 10]. Furthermore, routine use of x-rays is not advocated for diagnosis or assessment of knee OA [12, 15], yet people with OA expect imaging from their general practitioner [17, 18]. Receiving a diagnosis and explanation of OA that is based on x-ray reports and images may potentially contribute to stronger beliefs that surgery is required, and that exercise and physical activity is unsafe and should be avoided. To our knowledge, no previous RCTs have examined how a knee OA diagnosis and explanation based on x-ray affects consumer beliefs about OA management. Findings from this study will have implications for healthcare providers.

STUDY OBJECTIVES

Primary Objectives

1. To evaluate the effects of diagnosing and explaining knee OA based on x-rays (including showing a patient their x-ray images), on beliefs about knee pain management, compared to a diagnosis and explanation that does not involve using x-rays. We hypothesise that a diagnosis and explanation without x-ray will reduce beliefs that surgery is necessary and increase beliefs that exercise is helpful, compared to a diagnosis and explanation based on x-ray.
2. To evaluate the effects of showing a patient their x-ray images when explaining an x-ray report of knee OA, on beliefs about knee pain management, compared to not showing them their x-ray images. We hypothesise that a diagnosis and explanation without showing x-ray images will reduce beliefs that surgery is necessary and increase beliefs that exercise is helpful, compared to a diagnosis and explanation that involves showing x-ray images.

Secondary Objectives

To evaluate the effects of explaining an x-ray report of knee OA without showing a patient their x-ray images, on beliefs about OA management, compared to a diagnosis and explanation that does not involve sending the patient for x-ray.

STUDY DESIGN

Type of Study

Online prospective superiority three-arm RCT conducted in participants recruited from across Australia.

Study Design

An online three-arm superiority RCT will be conducted. The trial is designed according to SPIRIT (Standard Protocol Items: Recommendations for Interventional Trials) guidelines [21]. It will be prospectively registered on the Australia New Zealand Clinical Trials Registry and reported according to CONSORT statement and relevant extensions [22].

Number of Participants

609 participants with knee pain will be recruited*.*

Study sites

The study will be coordinated out of the University of Melbourne and participants will complete the trial (in a single online session) from their own home/workplace/location of their choice.

Expected Duration of Study

Anticipated participant recruitment start: April 2024

Anticipated data collection end: April 2024

Based on our previous similar study [2], which recruited 735 participants in four days through a similar mechanism, we anticipate recruitment for this RCT will take approximately 6 days.

Primary and Secondary Outcome Measures

**Table 1. Outcome measures collected in the trial at post-intervention**

| **Domain** | **Question** | **Scale** |
| --- | --- | --- |
| **Primary outcome measures** | | |
| Belief about joint replacement surgery | *Based on the video you have just watched, do you think joint replacement surgery (to replace the affected joint with an artificial joint) would be necessary for your hypothetical knee osteoarthritis at some stage?* | 11-point NRS ranging from 0=definitely not necessary to 10=definitely necessary |
| Belief about exercise and physical activity | *Based on the video you have just watched, do you think exercise and physical activity would be helpful to manage your hypothetical knee osteoarthritis?* | 11-point NRS ranging from 0=definitely not helpful to 10=definitely helpful |
| **Secondary outcome measures** | | |
| **Beliefs about treatment options** | | |
| Belief about safety of exercise | *Based on the video you have just watched, do you think exercise and physical activity could damage your hypothetical knee osteoarthritis?* | 11-point NRS ranging from 0=definitely would not damage it to 10=definitely would damage it |
| Belief about medication | *Based on the video you have just watched, do you think medication would help you manage your hypothetical knee osteoarthritis?* | 11-point NRS ranging from 0=definitely not helpful to 10=definitely helpful |
| **Level of concern** | | |
| Level of concern | *Based on the video you have just watched, how concerned would you be that your hypothetical knee osteoarthritis would get worse in the future?* | 11-point NRS ranging from 0=not concerned to 10=very concerned |
| **Fear of movement** | | |
| Brief Fear of Movement Scale [23] | *Based on the video you have just watched, and thinking about your hypothetical knee osteoarthritis, please answer the following questions:*   1. *I'm afraid that I might injure myself if I exercise* 2. *If I were to try to overcome it, my pain would increase* 3. *I am afraid that I might injure myself accidentally* 4. *Simply being careful that I do not make any unnecessary movements is the safest thing I can do to prevent my pain from worsening* 5. *It's really not safe for a person with a condition like mine to be physically active* 6. *I can't do all the things normal people do because it's too easy for me to get injured* | 4-point scale ranging “strongly disagree” (1), “disagree” (2), “agree” (3), and “strongly agree” (4)  Each item is scored 1-4. Scores are summed for an overall score ranging 6-24. Higher scores indicate greater fear of movement. |
| **Perceptions about healthcare providers** | | |
| Belief about orthopaedic surgeon | *Based on the video you have just watched, how much do you think an orthopaedic surgeon could help you with your hypothetical knee osteoarthritis?* | 11-point NRS ranging from 0=definitely could not help to 10=definitely could help |
| Belief about rheumatologist | *Based on the video you have just watched, how much do you think a rheumatologist could help you with your hypothetical knee osteoarthritis?* | 11-point NRS ranging from 0=definitely could not help to 10=definitely could help |
| Belief about physiotherapist | *Based on the video you have just watched, how much do you think a physiotherapist could help you with your hypothetical knee osteoarthritis?* | 11-point NRS ranging from 0=definitely could not help to 10=definitely could help |
| **Satisfaction** | | |
| Overall satisfaction | *Based on the video you have just watched, how satisfied would you be with this initial GP consultation about your hypothetical knee osteoarthritis?* | 11-point NRS ranging from 0=definitely not satisfied to 10=definitely satisfied |
| Satisfaction with information | *Based on the video you have just watched, how satisfied would you be with the information you received from the GP about your hypothetical knee osteoarthritis?* | 11-point NRS ranging from 0=definitely not satisfied to 10=definitely satisfied |
| Confidence | *Based on the video you have just watched, how confident would you be that the GP has provided an accurate diagnosis of your hypothetical knee osteoarthritis?* | 11-point NRS ranging from 0=not at all confident to 10=very confident |
| **Fidelity/process measures** | | |
| Whether watched allocated video or not | *Did you watch the video all the way through?* | Yes  No |
| Time spent watching video | Recorded by Qualtrics (time spent on page) | In minutes |
| **Baseline descriptive measures** | | |
| Gender | *Are you…* | Male  Female  Transgender male  Transgender female  Gender variant/non-conforming  Prefer not to say |
| Age | *What is your age?* | Self-reported in years |
| Ethnicity | *With what ethnicity do you most identify?* | Australian/New Zealand  Aboriginal and/or Torres Strait Islander  European  Asian  Other Oceania  North African & Middle Eastern  Sub-Saharan Africa  North American  South American  Prefer not to say  Other (please specify) |
| State living in | *What state do you live in?* | ACT  NSW  NT  QLD  SA  TAS  VIC  WA |
| Height | *What is your height?* | Self-reported in metres |
| Weight | *What is your weight?* | Self-reported in kilograms |
| Level of education | *What is the highest level of education you have completed?* | Primary school  High school  Trade or trade certificate  University or tertiary institute degree  Higher university degree (e.g. Masters, PhD)  Don’t know/unsure |
| Financial situation | *How would you describe your financial situation?* | Find it a strain to get by from week to week  Have to be careful with money  Able to manage without much difficulty  Quite comfortably off  Very comfortably off  Prefer not to answer |
| Level of exercise | *Do you currently participate in any type of regular exercise and/or physical activity (e.g. strengthening program, tennis, walking, cycling etc)* | No  Yes, 0-1 times per week  Yes, 2-3 times per week  Yes, 4-5 times per week  Yes, 6+ times per week |
| Medication use | *Do you regularly take pain relief medication for a musculoskeletal (i.e., bone/muscle/joint) condition?* | Yes  No |
| X-ray history | *Have you ever had an x-ray before?* | Yes  No  Not sure |
| Level of literacy for health information | *How easily can you read and understand written health information? I find it…* | 5-point Likert scale with response options:  Very difficult  Difficult  Neither easy nor difficult  Easy  Very easy |
| Painful joint | In which knee joint(s) have you experienced pain in the past 3 months? | Left knee only  Right knee only  Both knees |
| Pain | Select the number which indicates the average amount of pain felt over the PAST WEEK in your left knee/right knee/both knees. | NRS ranging from 0 (‘no pain’) to 10 (‘worst pain possible’) |
| Physical function | Select the number which indicates how much your left knee /right knee/both knees have interfered with your physical function over the PAST WEEK. | NRS ranging from 0 (‘no interference’) to 10 (‘maximal interference with function’) |

PARTICIPANT ENROLMENT AND RANDOMISATION

Recruitment

As we have done for our previous similar online RCTs [1-3]), we will engage a third party (the panel company, Cint) to facilitate study recruitment from their panel members across Australia. Cint is a consumer network for digital survey-based research (<https://www.cint.com/>). Our sample will comprise an equal proportion of people who have, and have not, experienced knee pain over the past 3 months.

Eligibility Criteria

Inclusion Criteria

Participants will be eligible for the study if they meet the following inclusion criteria:

1. Adults aged 45 years or over;
2. Currently living in Australia;
3. Have, and have not, experienced activity-related knee joint pain in the last 3 months; and
4. Have never consulted a healthcare professional for their knee pain.

Exclusion Criteria

1. Inability to understand or read English

Informed Consent Process

All potential participants will receive written information about the purpose of the study at the start of the electronic survey. According to the latest revision of the World Medical Association Declaration of Helsinki, informed consent will be obtained from all participants by indicating that they consent (by ticking a box in the survey) after understanding the information delivered.

Enrolment and Randomisation Procedures

Participants will be automatically enrolled into the study once they pass the screening questions and consent to participate. After completing demographic measures, participants will be automatically randomised to one of the three groups (1. *clinical diagnosis and explanation without x-ray; 2. diagnosis and explanation based on x-ray findings (but without being shown their x-ray images); or 3. diagnosis and explanation based on x-ray findings (and showing their x-ray images)*) by the Qualtrics software (using the “randomiser” feature set to evenly present elements at a 1:1:1 ratio), stratified by whether or not they have knee pain.

Blinding Arrangements

Although participants will obviously be aware of the elements of their randomised hypothetical GP diagnosis and explanation video (by virtue of having watched the video), participants will be blinded to study hypotheses and the alternative conditions by a process of limited disclosure. Participants will simply be told that the study compares three different types of knee pain information provided by a GP, without disclosing the specific elements of each. Research staff managing the trial database will be blinded. Statistical analyses will be performed blinded*.*

Participant Withdrawal

As participation in this study is voluntary, participants may withdraw from the study by abandoning the survey at any time. Consistent with our prior RCTs [1-3], only data from complete surveys will be used in analysis.

Trial Closure

The survey will take approximately 20 minutes for each participant to complete. Once the required sample size has completed the survey, the survey will close and the trial will be considered closed.

STUDY PROCEDURES SCHEDULE

Demographic questions

***Clinical diagnosis and explanation without sending for an x-ray***

**Follow-up**

**Intervention**

**Randomisation**

Primary and secondary outcome measures collected

Randomisation by Qualtrics

***Diagnosis and explanation based on x-ray (showing the x-ray images)***

***Diagnosis and explanation based on x-ray (without showing the x-ray images)***

**Figure 1. Participant flow through the trial survey**

DATA COLLECTION

The entire trial will be administered in one single online survey completed in one session (Figure 1). All participants will complete outcome measures online via Qualtrics immediately after the intervention. It is anticipated the entire online session will take 20 minutes (to complete questionnaires and watch allocated video)

INTERVENTIONS

Participants will be asked to read the following hypothetical scenario:

“*Imagine that one of your knees has been painful for about six months. At first, the pain was on-and-off, but lately has been getting worse and it is now bothering you on most days. You feel the knee pain during walking and using the stairs, but are unsure what caused the pain to start. Imagine that you have made an appointment with a general practitioner (GP) to find out what is wrong with your knee.*”

Participants will then be randomised to one of three groups, where they will each be provided with a different video of a GP (Dr Ewald) diagnosing and explaining knee OA. Video scripts were created by the research team (including a practicing GP (Dr Ewald) and a consumer with lived experience of knee OA (Ms Capewell)). The three groups are:

1. *Clinical diagnosis and explanation without being sent for x-ray*;
2. *Diagnosis and explanation based on x-ray findings (but without showing x-ray images)*;
3. *Diagnosis and explanation based on x-ray findings (and showing x-ray images).*

**GROUP 1: CLINICAL DIAGNOSIS AND EXPLANATION WITHOUT BEING SENT FOR X-RAY**

Participant instructions: Please watch the following video, imagining that this is a GP and this is what they tell you after you’ve described your knee pain and the GP has examined your knee:

“*The most common cause of knee pain in people aged 45 and over is osteoarthritis. Based on your age and the nature of your symptoms, such as having pain with activity like you have described, I think osteoarthritis is the cause of your knee pain. Special tests- such as an x-ray- aren’t recommended unless we need to rule out another cause of your knee pain. We would only do that if you had unusual symptoms – which you don’t have. Also, x-rays don’t tell us what will happen with your knee in the future, and we can recommend treatments for you without needing an x-ray. X-rays also expose you to radiation, and it’s best to minimise your exposure.*

*Osteoarthritis is a condition of the whole knee, including surrounding cartilage, bones, ligaments, and muscles. Initially, changes occur to some of the structures in the knee. This may be due to previous injuries, genetics, or the type of work you’ve done. The body will then try to repair these changes. While these repair processes can lead to further joint changes, they often keep the knee working normally. Some people have lots of changes in their knee joint, but this doesn’t necessarily mean they have more knee pain.*

*For most people, osteoarthritis won’t get progressively worse. In fact, only about a third of people will get worse over time. There are treatments that can provide pain relief and allow you to get on with your life. Usually, treatment includes an exercise program tailored to your condition and ability, medicines like paracetamol or non-steroidal anti-inflammatory drugs, and weight loss if necessary. Some people may need joint replacement surgery if those treatments aren’t effective.*

*I'm sure you have many questions about your knee pain after hearing all that. I'm happy to answer any questions you have.”*

**GROUP 2: DIAGNOSIS AND EXPLANATION BASED ON X-RAY FINDINGS (BUT WITHOUT SHOWING X-RAY IMAGES)**

Participant instructions: Please watch the following video, imagining that this is a GP and this is what they tell you after you’ve described your knee pain and the GP has examined your knee:

*“It sounds like you could have knee osteoarthritis – I’m going to send you for x-rays of your knee to confirm what is going on in your joint.”*

*text shown on screen: *imagine you have gone and had x-rays of your knee and have now returned to the GP for the results**

*“I have the report from the x-rays of your knee, which show you do have osteoarthritis, like I suspected. The report states that, in your painful knee, there is reduction in the joint space – which means that the area of the knee joint between the bones is narrower, showing that the cartilage is thinner. The report also states that there are osteophytes - or bony spurs - that are growing around the edges of the joint.*

*Osteoarthritis is a condition of the whole knee, including surrounding cartilage, bones, ligaments, and muscles. Initially, changes occur to some of the structures in the knee. This may be due to previous injuries, genetics, or the type of work you’ve done. The body will then try to repair these changes. While these repair processes can lead to further joint changes, they often keep the knee working normally. Some people have lots of changes in their knee joint, but this doesn’t necessarily mean they have more knee pain.*

*For most people, osteoarthritis won’t get progressively worse. In fact, only about a third of people will get worse over time. There are treatments that can provide pain relief and allow you to get on with your life. Usually, treatment includes an exercise program tailored to your condition and ability, medicines like paracetamol or non-steroidal anti-inflammatory drugs, and weight loss if necessary. Some people may need joint replacement surgery if those treatments aren’t effective.*

*I'm sure you have many questions about your knee pain after hearing all that. I'm happy to answer any questions you have.”*

**GROUP 3: DIAGNOSIS AND EXPLANATION BASED ON X-RAY FINDING (AND SHOWING X-RAY IMAGES)**

Participant instructions: Please watch the following video, imagining that this is a GP and this is what they tell you after you’ve described your knee pain and the GP has examined your knee:

*“It sounds like you could have knee osteoarthritis – I’m going to send you for x-rays of your knee to confirm what is going on in your joint.”*

*text shown on screen: *imagine you have gone and had x-rays of your knee and have now returned to the GP for the results**

*“Here are the x-rays of your knee (**show **IMAGE A** on screen**), which show you do have osteoarthritis, like I suspected. As you can see here (**show **IMAGE B** on screen**), this is your normal knee – you can see the nice space between the bones in your joint indicating healthy cartilage. This (**show **IMAGE C** on screen**) is your painful knee, which shows you have osteoarthritis. This area of the knee joint between the bones is narrower, showing where the cartilage is thinner. Over here (**show **IMAGE D** on screen**), you can see some osteophytes- or bony spurs- that are growing around the edges of the joint.*

*Osteoarthritis is a condition of the whole knee, including surrounding cartilage, bones, ligaments, and muscles. Initially, changes occur to some of the structures in the knee. This may be due to previous injuries, genetics, or the type of work you’ve done. The body will then try to repair these changes. While these repair processes can lead to further joint changes, they often keep the knee working normally. Some people have lots of changes in their knee joint, but this doesn’t necessarily mean they have more knee pain.*

*For most people, osteoarthritis won’t get progressively worse. In fact, only about a third of people will get worse over time. There are treatments that can provide pain relief and allow you to get on with your life. Usually, treatment includes an exercise program tailored to your condition and ability, medicines like paracetamol or non-steroidal anti-inflammatory drugs, and weight loss if necessary. Some people may need joint replacement surgery if those treatments aren’t effective.*

*I'm sure you have many questions about your knee pain after hearing all that. I'm happy to answer any questions you have.”*


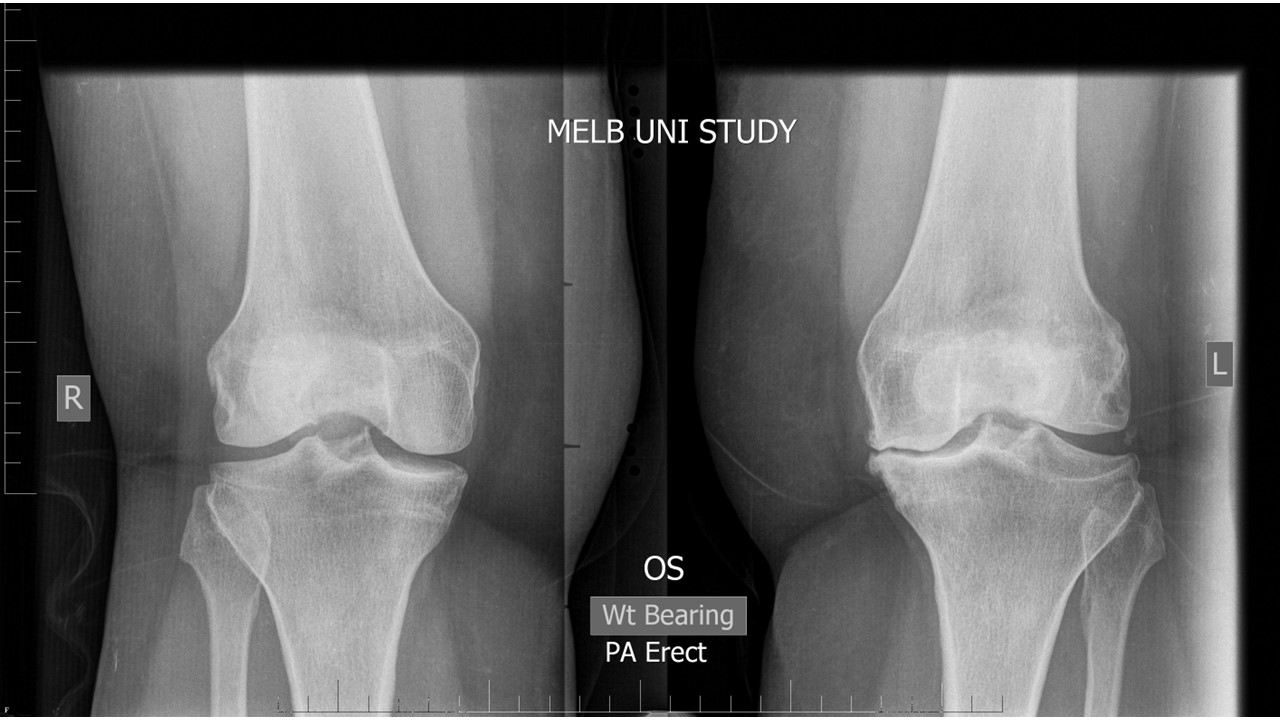


**IMAGE A**


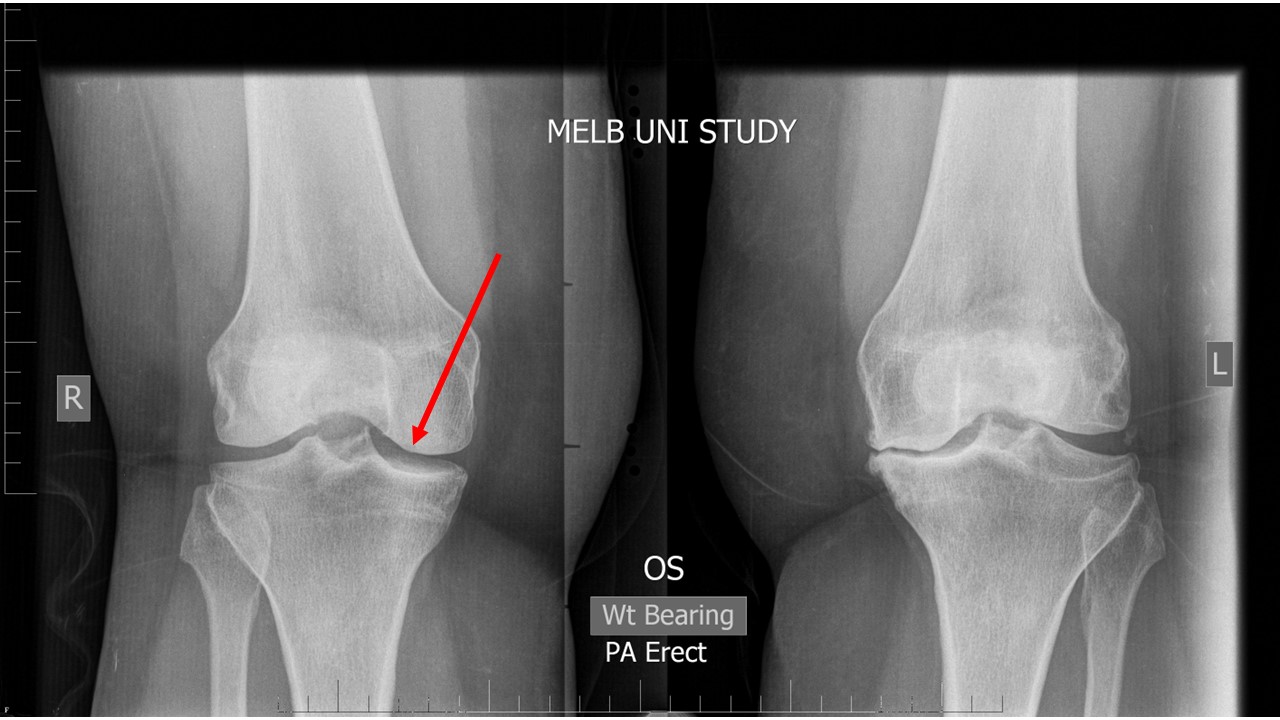


**IMAGE B**


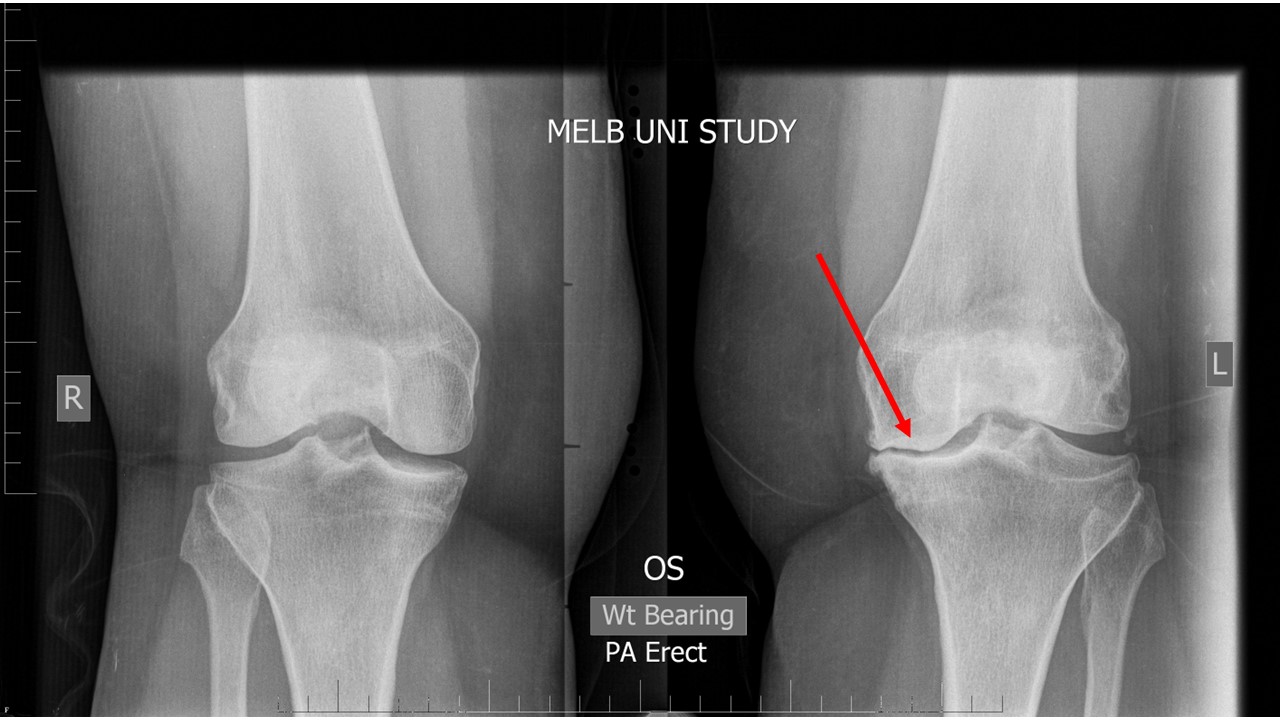


**IMAGE C**

**
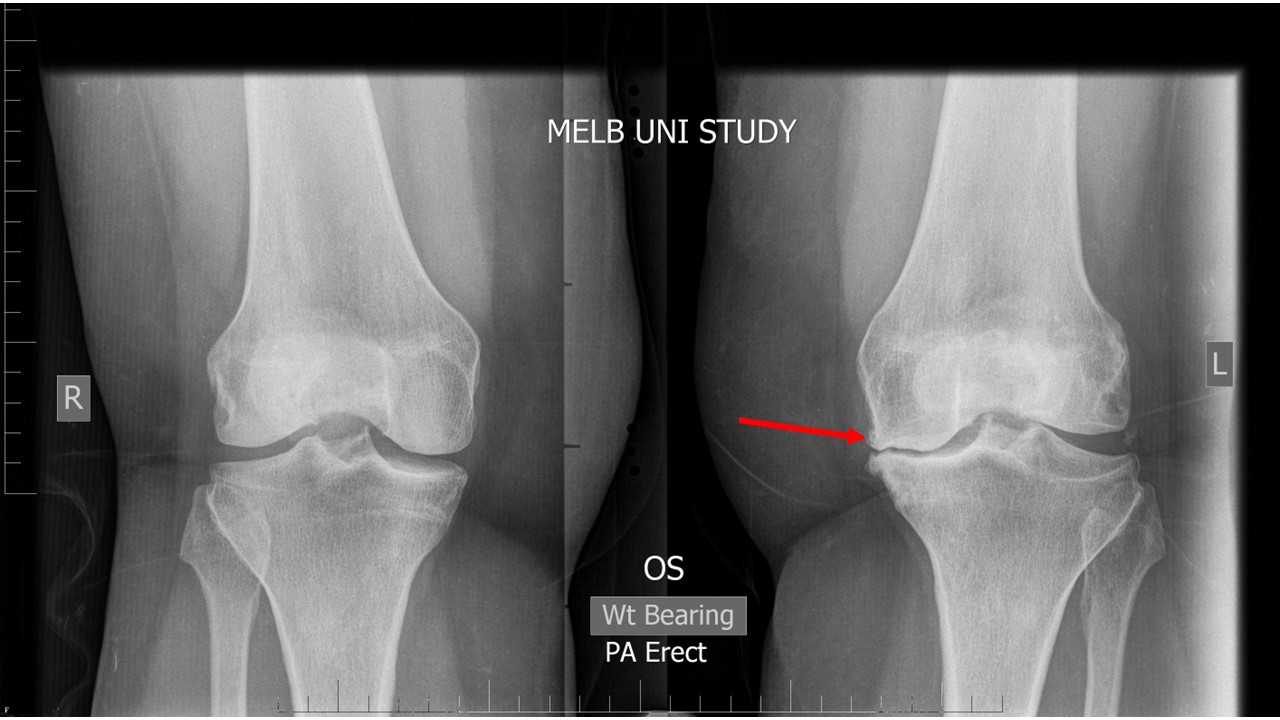
**

**IMAGE D**

ADVERSE EVENT REPORTING

Not relevant as this is an educational intervention hypothesised to affect beliefs and knowledge, rather than clinical outcomes.

STATISTICAL METHODS

Sample Size Estimation

The sample size is based on detecting a between-group difference of 1 NRS unit for both primary outcomes, beliefs about joint replacement surgery and beliefs about exercise and physical activity (based on data from a previous trial [3]). Assuming a between-participant standard deviation of 3 NRS units for both outcomes [3], to achieve 80% power, with a two-sided significance level of 0.0125 (conservative Bonferroni correction for two primary outcomes and two primary pair-wise comparisons), and accounting for no attrition [1], we require 203 participants per arm, or a total of 609 participants.

Statistical Analysis Plan

A statistical analysis plan will be finalised prior to data analysis and published on our centre’s website. A biostatistician will analyse blinded data. As in our prior online RCTs [1-3], we will use complete case analyses. Briefly, primary and secondary outcomes post-intervention will be analysed using linear regression models. Results will be presented as estimated mean differences between groups, with two-sided 95% confidence intervals and p-values (multiplicity adjusted for primary outcomes).

DATA MANAGEMENT

Data Collection & Storage

**Identifiable data:**

- No identifiable data (names, contact details) will be collected in the trial. If participants opt into receiving a report of findings at completion of the study, they will be automatically redirected to a new survey to provide their email address to ensure anonymity of their data provided in the trial.

**Re-identifiable/coded data:**

- The survey will be completed electronically. Data will be stored in Qualtrics, accessible only to the researchers by password protection. Data from within Qualtrics will be exported to Microsoft Excel and other statistical packages used by the researchers for analyses and stored securely on password-protected University servers.
- All computer files will be stored on secure and backed-up servers, accessibly only to the researchers using a password.

Data Confidentiality

No information which could lead to the identification of a participant will be included in the dissemination of results.

Study Record Retention

Data will be retained for 15 years after final publication consistent with clinical trial recommendations outlined in section 2.1.1 of the National Health and Medical Research Council’s “Australian Code for the Responsible Conduct of Research”.

ADMINISTRATIVE ASPECTS

The trial will be prospectively registered (Australia New Zealand Clinical Trials Register).

Independent HREC approval

This study requires approval by the University of Melbourne Human Research Ethics Committee (HREC).

Participant reimbursement

Participants will not receive any reimbursement for completing the trial from researchers. As part of their membership with Cint, they will receive a payment from Cint for completing the survey, which The University of Melbourne has no involvement in.

Financial disclosure and conflicts of interest

n/a

USE OF DATA AND PUBLICATIONS POLICY

The trial will be published in a musculoskeletal, rheumatology or general medical journal.

Statistical code may be made available from Dr Lawford, upon reasonable request from individual researchers.

The results of the trial will also be disseminated through avenues such as conference presentations, professional organisations, media, social media and consumer organisations.

REFERENCES

1. Lawford, B., et al., *Comparing effects of knee osteoarthritis educational information, with and without pathoanatomical content, on consumer osteoarthritis management beliefs: An online randomised controlled trial.* Journal of Orthopaedic & Sports Physical Therapy, 2022. **Accepted Nov 2022**.

2. Lawford, B., et al., *Effect of information content and general practitioner recommendation on treatment beliefs and intentions for knee osteoarthritis: An online multi-arm randomised controlled trial.* ACR Open Rheumatology, 2022. **Accepted Nov 2022**.

3. Haber, T., et al., *Effects of Hip Pain Diagnostic Labels and Their Explanations on Beliefs About Hip Pain and How to Manage It: An Online Randomized Controlled Trial.* Journal of Orthopaedic & Sports Physical Therapy, 2023. **53**(11): p. 673-684.

4. Cross, M., et al., *The global burden of hip and knee osteoarthritis: estimates from the global burden of disease 2010 study.* Annals of the Rheumatic Diseases, 2014. **73**(7): p. 1323-30.

5. Kolasinski, S.L., et al., *2019 American College of Rheumatology/Arthritis Foundation Guideline for the Management of Osteoarthritis of the Hand, Hip, and Knee.* Arthritis & Rheumatology, 2020. **72**(2): p. 149-162.

6. Bannuru, R.R., et al., *OARSI guidelines for the non-surgical management of knee, hip, and polyarticular osteoarthritis.* Osteoarthritis and Cartilage, 2019. **27**(11): p. 1578-1589.

7. The Royal Australian College of General Practitioners, *Guideline for the management of knee and hip osteoarthritis*. 2018, RACGP: East Melbourne, VIC.

8. National Institute for Health and Care Excellence, *Osteoarthritis in over 16s: diagnosis and management*, National Institute for Health and Care Excellence, Editor. 2022: London.

9. Baumbach, L., et al., *Patients with osteoarthritis are least likely to receive lifestyle advice compared with patients with diabetes and hypertension: a national health survey study from Denmark.* Osteoarthritis and Cartilage Open, 2020. **2**(3): p. 100067.

10. Bennell, K.L., et al., *Trends in management of hip and knee osteoarthritis in general practice in Australia over an 11-year window: a nationwide cross-sectional survey.* The Lancet Regional Health-Western Pacific, 2021. **12**: p. 100187.

11. National Institute for Health and Care Excellence, *Osteoarthritis - Care and management, clinical guideline CG177*, National Institute for Health and Care Excellence, Editor. 2014: London.

12. Zhang, W., et al., *EULAR evidence-based recommendations for the diagnosis of knee osteoarthritis.* Ann Rheum Dis, 2010. **69**(3): p. 483-9.

13. Bunzli, S., et al., *Misconceptions and the acceptance of evidence-based nonsurgical interventions for knee osteoarthritis. A qualitative study.* Clinical Orthopaedics and Related Research, 2019. **477**(9): p. 1975.

14. Steenkamp, W., et al., *The correlation between clinical and radiological severity of osteoarthritis of the knee.* SICOT-J, 2022. **8**.

15. Hannan, M.T., D.T. Felson, and T. Pincus, *Analysis of the discordance between radiographic changes and knee pain in osteoarthritis of the knee.* The Journal of rheumatology, 2000. **27**(6): p. 1513-1517.

16. Nalamachu, S., et al., *Pain severity and healthcare resource utilization in patients with osteoarthritis in the United States.* Postgraduate Medicine, 2021. **133**(1): p. 10-19.

17. Darlow, B., et al., *Living with osteoarthritis is a balancing act: an exploration of patients’ beliefs about knee pain.* BMC Rheumatology, 2018. **2**(1): p. 1-9.

18. Egerton, T., et al., *General practitioners’ views on managing knee osteoarthritis: a thematic analysis of factors influencing clinical practice guideline implementation in primary care.* BMC Rheumatology, 2018. **2**(1): p. 30.

19. Haber, T., et al., *Peoples' beliefs about their chronic hip pain and its care: a systematic review of qualitative studies. "I'm just getting old and breaking down".* Pain, 2023. **164**(5): p. 926-947.

20. Karran, E.L., et al., *The impact of choosing words carefully: an online investigation into imaging reporting strategies and best practice care for low back pain.* PeerJ, 2017. **5**: p. e4151.

21. Chan, A.W., et al., *SPIRIT 2013 statement: defining standard protocol items for clinical trials.* Annals of Internal Medicine, 2013. **158**(3): p. 200-7.

22. Moher, D., et al., *CONSORT 2010 explanation and elaboration: updated guidelines for reporting parallel group randomised trials.* BMJ, 2010. **340**: p. c869.

23. Shelby, R.A., et al., *Brief Fear of Movement Scale for osteoarthritis.* Arthritis Care & Research, 2012. **64**(6): p. 862-71.
