# Supplementary material for: Effects of X-ray–based diagnosis and explanation of knee osteoarthritis on patient beliefs about osteoarthritis management: A randomised clinical trial
Source: PLoS Med. 2025 Feb 4;22(2):e1004537. doi: 10.1371/journal.pmed.1004537 (PMC11838874; doi:10.1371/journal.pmed.1004537)
Supplement: S3 Appendix — (DOCX) [file pmed.1004537.s003.docx]

# S3 Appendix. Statistical Analysis Plan


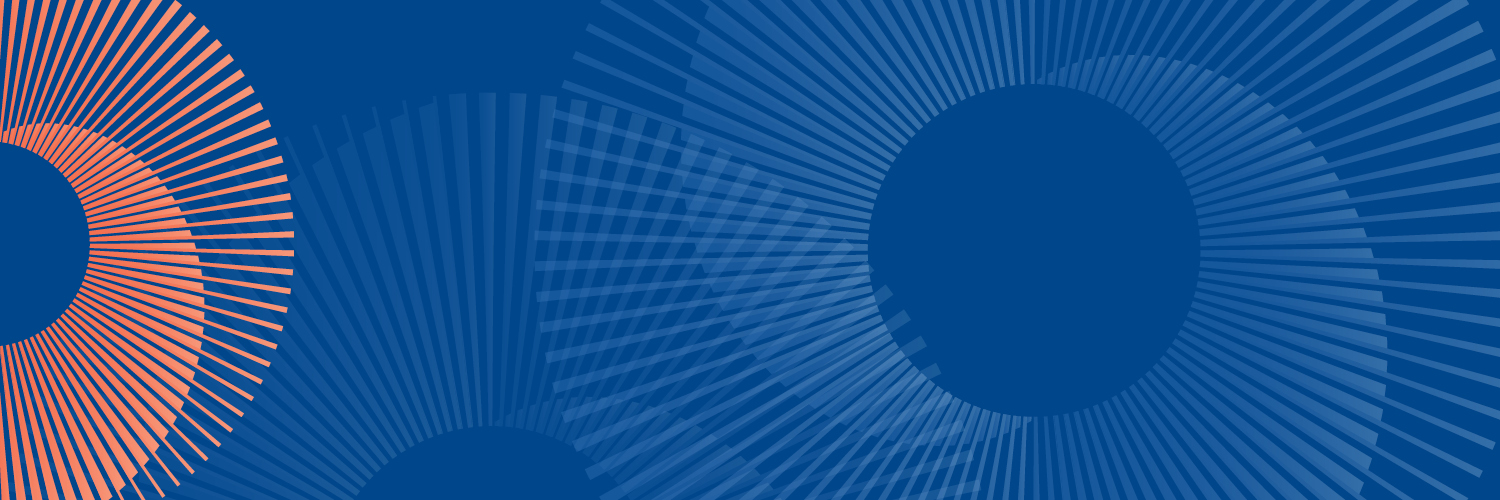


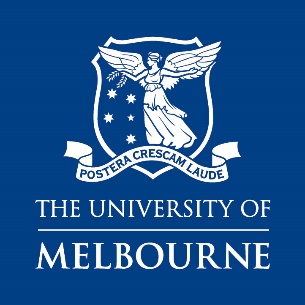


**SOP No.** 1

**Date:** October 2021

**Review Date:** October 2022

**Version No.** 1

Authorised by: MISCH Director

**[MSPGH/Dept of Epidemiology & Biostatistics]**

**MISCH STANDARD OPERATING PROCEDURE**

**Statistical Analysis Plan**

Statistical Analysis Plan (Short)

Effects of a radiographic diagnosis and explanation of knee osteoarthritis on consumer beliefs about knee pain management: an online randomised controlled trial.

Version: 3.0

Authors: Ms Peixuan Li and Dr Anurika De Silva, Biostatisticians

*Methods and Implementation Support for Clinical and Health (MISCH) research Hub,* *Faculty of Medicine, Dentistry and Health Sciences, University of Melbourne*

Dr Belinda Lawford, Lead investigator

*CHESM, University of Melbourne*

Date: 23/01/2025

Author/s of template: Ms Sabine Braat (Deputy Head MISCH Biostatistics), University of Melbourne (Template adapted from www.pfizer.com)

Date of template: 25/10/2021

1 Introduction

| Objectives | **1** **Primary Objectives**   1. To determine whether a hypothetical diagnosis and explanation of knee OA that does not involve using x-rays can improve i) belief about joint replacement surgery or ii) belief about exercise and physical activity, compared to a diagnosis and explanation of knee OA based on x-rays (and showing a patient their x-ray images) (primary comparison 1). 2. To determine whether a hypothetical diagnosis and explanation of knee OA based on x-rays (and not showing the patient their x-ray images) can improve i) belief about joint replacement surgery or ii) belief about exercise and physical activity, compared to a diagnosis and explanation of knee OA based on x-rays (and showing a patient their x-ray images) (primary comparison 2). 3. **Secondary Objectives** 4. To determine whether a hypothetical diagnosis and explanation of knee OA that does not involve using x-rays can improve i) belief about joint replacement surgery or ii) belief about exercise and physical activity, compared to a diagnosis and explanation of knee OA based on x-rays (and not showing a patient their x-ray images) (secondary comparison). 5. To compare i) belief exercise would damage knee, ii) belief medication is helpful, iii) level of concern, iv) fear of movement, v) belief about helpfulness of orthopaedic surgeon, vi) belief about helpfulness of rheumatologist, vii) belief about helpfulness of physiotherapist, viii) overall satisfaction with consultation, ix) satisfaction with information provided, x) confidence in accuracy of diagnosis, across the two primary comparisons and the secondary comparison. 6. To explore whether a history of knee pain in the last 3 months moderates the effect of the interventions on the two primary outcomes for each primary comparison. |
| --- | --- |
| Study Design | Three-arm, parallel groups superiority randomised controlled trial (RCT). |
| Planned Sample Size | 609 participants. |
| Study Procedures | The entire trial will be administered in one single online survey. Participants will initially complete screening questions to determine eligibility. Eligible participants will then be asked to imagine a hypothetical scenario where they have made an appointment with a GP to find out what is wrong with their painful knee. Participants will then be automatically randomised by Qualtrics to one of three groups, all of whom will be asked to watch a pre-recorded video on their screen where they receive a hypothetical knee OA diagnosis from the GP:   1. Clinical diagnosis and explanation without being sent for x-ray: the video will show the GP providing a clinical diagnosis of knee OA based on their age and symptoms, including an explanation of why x-rays are not necessary for OA diagnosis. The GP will then explain what OA is. 2. Diagnosis and explanation based on x-ray findings (but without showing the x-ray images): the video will show the GP ordering an x-ray for the participant, summarising the x-ray report (but not showing the participant the x-ray images), and providing a diagnosis of knee OA. The GP will then explain what OA is. 3. Diagnosis and explanation based on x-ray (and showing the x-ray images): the video will show the GP ordering an x-ray for the participant, then summarising the x-ray report and showing them the x-ray images, and providing a diagnosis of knee OA. The GP will then explain what OA is.   All participants will complete outcome measures immediately after watching their allocated video. |
| Duration of the study | Anticipated participant recruitment start: May 2024  Anticipated data collection end: June 2024  Based on our previous similar study [1], which recruited 735 participants in four days through a similar mechanism, we anticipate recruitment for this RCT will take approximately 6 days. |

Demographic questions

***Clinical diagnosis and explanation without sending for an x-ray***

**Follow-up**

**Intervention**

**Randomisation**

Primary and secondary outcome measures collected

Randomisation by Qualtrics

***Diagnosis and explanation based on x-ray (showing the x-ray images)***

***Diagnosis and explanation based on x-ray (without showing the x-ray images)***

**Figure 1. Participant flow through the trial survey**

2 Data Source

Participant recruitment will be managed by an external company, Cint Pty Ltd. Data security will be managed according to their data privacy and security protocols.

Data collection will be managed wholly online through Qualtrics, an electronic data capture system. Qualtrics is a secure web application for building and managing research questionnaires and databases. The University of Melbourne holds a licence for staff use of Qualtrics and surveys and associated data will only be accessible via secure login by the study research staff.

*Re-identifiable/coded data*

Questionnaires will be completed electronically and anonymously, with no identifying information being recorded. Electronic data will be stored in the Qualtrics website, accessible only to the study research staff by password protection. Data from within Qualtrics will eventually be exported to Microsoft Excel and other statistical packages used by the researchers for analyses and stored securely on password-protected servers accessible only to the study research staff.

3 Analysis Objectives

3.1 Primary objectives

The primary objectives are:

1. To determine whether a hypothetical diagnosis and explanation of knee OA that does not involve using x-rays can improve i) belief about joint replacement surgery or ii) belief about exercise and physical activity, compared to a diagnosis and explanation of knee OA based on x-rays (and showing a patient their x-ray images). We hypothesise that a hypothetical diagnosis and explanation of knee OA that does not involve using x-rays improves i) belief about joint replacement surgery or ii) belief about exercise and physical activity, compared to a diagnosis and explanation of knee OA based on x-rays (and showing a patient their x-ray images) (primary comparison 1).
2. To determine whether a hypothetical diagnosis and explanation of knee OA based on x-rays (and not showing the patient their x-ray images) can improve i) belief about joint replacement surgery or ii) belief about exercise and physical activity, compared to a diagnosis and explanation of knee OA based on x-rays (and showing a patient their x-ray images). We hypothesis that a hypothetical diagnosis and explanation of knee OA based on x-rays (and not showing the patient their x-ray images) improves i) belief about joint replacement surgery or ii) belief about exercise and physical activity, compared to a diagnosis and explanation of knee OA based on x-rays (and showing a patient their x-ray images) (primary comparison 2).

3.2 Secondary objectives

The secondary objectives are:

1. To determine whether a hypothetical diagnosis and explanation of knee OA that does not involve using x-rays can improve i) belief about joint replacement surgery or ii) belief about exercise and physical activity, compared to a diagnosis and explanation of knee OA based on x-rays (and not showing a patient their x-ray images) (secondary comparison).
2. To compare i) belief exercise would damage knee, ii) belief medication is helpful, iii) level of concern, iv) fear of movement, v) belief about helpfulness of orthopaedic surgeon, vi) belief about helpfulness of rheumatologist, vii) belief about helpfulness of physiotherapist, viii) overall satisfaction with consultation, ix) satisfaction with information provided, x) confidence in accuracy of diagnosis, across the two primary comparisons and the secondary comparison.
3. To explore whether a history of knee pain in the last 3 months moderates the effect of the interventions on the two primary outcomes for each primary comparison.

4 Analysis sets/Populations/Subgroups

Inclusion Criteria

Participants will be eligible for the study if they meet the following inclusion criteria:

1. Adults aged 45 years or over;
2. Currently living in Australia;
3. Either have, or have not, experienced activity-related knee joint pain over the last 3 months; and
4. Have never consulted a healthcare professional for chronic knee pain.

*Exclusion Criteria*

Participants will be ineligible for the study if they have:

1. Inability to understand or read English.

5 Endpoints and Covariates

All variables are listed in Appendix 1 and the definition of the variables can be found in Appendix 2. Outcome measures are provided in the table below.

| **Domain** | **Question** | **Scale** |
| --- | --- | --- |
| **Primary outcome measures** | | |
| Belief about joint replacement surgery | *Based on the video you have just watched, do you think joint replacement surgery (to replace the affected joint with an artificial joint) would be necessary for your hypothetical knee osteoarthritis at some stage?* | 11-point Numerical rating scale (NRS) ranging from 0=definitely not necessary to 10=definitely necessary |
| Belief about exercise and physical activity | *Based on the video you have just watched, do you think exercise and physical activity would be helpful to manage your hypothetical knee osteoarthritis?* | 11-point NRS ranging from 0=definitely not helpful to 10=definitely helpful |
| **Secondary outcome measures** | | |
| **Beliefs about treatment options** | | |
| Belief about safety of exercise | *Based on the video you have just watched, do you think exercise and physical activity could damage your hypothetical knee osteoarthritis?* | 11-point NRS ranging from 0=definitely would not damage it to 10=definitely would damage it |
| Belief about medication | *Based on the video you have just watched, do you think medication would help you manage your hypothetical knee osteoarthritis?* | 11-point NRS ranging from 0=definitely not helpful to 10=definitely helpful |
| **Level of concern** | | |
| Level of concern | *Based on the video you have just watched, how concerned would you be that your hypothetical knee osteoarthritis would get worse in the future?* | 11-point NRS ranging from 0=not concerned to 10=very concerned |
| **Fear of movement** | | |
| Brief Fear of Movement Scale [2] | *Based on the video you have just watched, and thinking about your hypothetical knee osteoarthritis, please answer the following questions:*   1. *I'm afraid that I might injure myself if I exercise* 2. *If I were to try to overcome it, my pain would increase* 3. *I am afraid that I might injure myself accidentally* 4. *Simply being careful that I do not make any unnecessary movements is the safest thing I can do to prevent my pain from worsening* 5. *It's really not safe for a person with a condition like mine to be physically active* 6. *I can't do all the things normal people do because it's too easy for me to get injured* | 4-point scale ranging “strongly disagree” (1), “disagree” (2), “agree” (3), and “strongly agree” (4)  Each item is scored 1-4. Scores are summed for an overall score ranging 6-24. Higher scores indicate greater fear of movement. |
| **Perceptions about healthcare providers** | | |
| Belief about orthopaedic surgeon | *Based on the video you have just watched, how much do you think an orthopaedic surgeon could help you with your hypothetical knee osteoarthritis?* | 11-point NRS ranging from 0=definitely could not help to 10=definitely could help |
| Belief about rheumatologist | *Based on the video you have just watched, how much do you think a rheumatologist could help you with your hypothetical knee osteoarthritis?* | 11-point NRS ranging from 0=definitely could not help to 10=definitely could help |
| Belief about physiotherapist | *Based on the video you have just watched, how much do you think a physiotherapist could help you with your hypothetical knee osteoarthritis?* | 11-point NRS ranging from 0=definitely could not help to 10=definitely could help |
| **Satisfaction** | | |
| Overall satisfaction | *Based on the video you have just watched, how satisfied would you be with this initial GP consultation about your hypothetical knee osteoarthritis?* | 11-point NRS ranging from 0=definitely not satisfied to 10=definitely satisfied |
| Satisfaction with information | *Based on the video you have just watched, how satisfied would you be with the information you received from the GP about your hypothetical knee osteoarthritis?* | 11-point NRS ranging from 0=definitely not satisfied to 10=definitely satisfied |
| Confidence | *Based on the video you have just watched, how confident would you be that the GP has provided an accurate diagnosis of your hypothetical knee osteoarthritis?* | 11-point NRS ranging from 0=not at all confident to 10=very confident |
| **Fidelity/process measures** | | |
| Whether watched allocated video or not | *Did you watch the video all the way through?* | Yes  No |
| Time spent watching video | Recorded by Qualtrics (time spent on page) | In minutes |
| **Baseline descriptive measures** | | |
| Gender | *Are you…* | Male  Female  Transgender male  Transgender female  Gender variant/non-conforming  Prefer not to say |
| Age | *What is your age?* | Self-reported in years |
| Ethnicity | *With what ethnicity do you most identify?* | Australian/New Zealand  Aboriginal and/or Torres Strait Islander  European  Asian  Other Oceania  North African & Middle Eastern  Sub-Saharan Africa  North American  South American  Prefer not to say  Other (please specify) |
| State living in | *What state do you live in?* | ACT  NSW  NT  QLD  SA  TAS  VIC  WA |
| Height | *What is your height?* | Self-reported in metres |
| Weight | *What is your weight?* | Self-reported in kilograms |
| Level of education | *What is the highest level of education you have completed?* | Primary school  High school  Trade or trade certificate  University or tertiary institute degree  Higher university degree (e.g. Masters, PhD)  Don’t know/unsure |
| Financial situation | *How would you describe your financial situation?* | Find it a strain to get by from week to week  Have to be careful with money  Able to manage without much difficulty  Quite comfortably off  Very comfortably off  Prefer not to answer |
| Level of exercise | *Do you currently participate in any type of regular exercise and/or physical activity (e.g. strengthening program, tennis, walking, cycling etc)* | No  Yes, 0-1 times per week  Yes, 2-3 times per week  Yes, 4-5 times per week  Yes, 6+ times per week |
| Medication use | *Do you regularly take pain relief medication for a musculoskeletal (i.e., bone/muscle/joint) condition?* | Yes  No |
| X-ray history | *Have you ever had an x-ray before?* | Yes  No  Not sure |
| Level of literacy for health information | *How easily can you read and understand written health information? I find it…* | 5-point Likert scale with response options:  Very difficult  Difficult  Neither easy nor difficult  Easy  Very easy |
| Knee pain | Have you had activity-related knee joint pain (e.g., pain while walking up the stairs or bending down) over the last 3 months? | Yes  No |
| Painful joint | In which knee joint(s) have you experienced pain in the past 3 months? | Left knee only  Right knee only  Both knees |
| Pain | Select the number which indicates the average amount of pain felt over the PAST WEEK in your left knee/right knee/both knees. | NRS ranging from 0 (‘no pain’) to 10 (‘worst pain possible’) |
| Physical function | Select the number which indicates how much your left knee /right knee/both knees have interfered with your physical function over the PAST WEEK. | NRS ranging from 0 (‘no interference’) to 10 (‘maximal interference with function’) |

6 Handling of Missing Values and Other Data Conventions

If missing data are present, an appendix table will provide summaries of baseline characteristics and baseline levels of primary and secondary outcomes (where measured) between two groups: those participants who provide both primary outcomes post-intervention, and those participants who are missing either or both primary outcomes. If less than 5% of both primary outcomes are missing, analyses will be performed on complete case data. If greater than 5% of either primary outcome is missing, multiply imputed data will be used for the primary analysis. Missing outcomes will be imputed using chained equations with predictive mean matching and five nearest neighbours for continuous outcomes. Imputation models for continuous outcomes post-intervention will include all primary and secondary outcomes at both baseline and post-intervention, along with age, gender, BMI, education level, state, ethnicity, financial situation, level of exercise, knee pain, regular pain relief for musculoskeletal condition, and health literacy. Data will be imputed for each treatment group separately. The number of imputed data sets created will be based on the percentage of patients in the sample with missing outcome data (e.g., 15 imputed datasets if 15% of participants have missing data). Estimates from the imputed datasets will be combined using Rubin’s rules [3].

7 Statistical Methodology

7.1 Statistical Procedures

A biostatistician (Ms Peixuan Li, supervised by Dr Anurika De Silva) will analyse de-identified data using Stata/SE 17.0 (StataCorp LLC, College Station, TX, USA). Baseline data (age, gender, ethnicity, state/territory, height, weight, body mass index, highest level of education, financial situation, level of exercise, medication use, X-ray history, level of literacy for health information, painful joint, average level of knee pain, and physical function) will be summarised by intervention group as mean (standard deviation, SD), median (interquartile range, IQR) or n (%) as appropriate. Comparative analyses between groups will be performed using intention-to-treat according to the group to which the participant was randomised irrespective of whether they adhered to the intervention by reading the information. The number included in each analysis will be reported.

7.1.1 Primary outcomes

Separate linear regression models will be used for each primary outcome post-intervention, i) belief about joint replacement surgery and ii) belief about exercise and physical activity, to estimate the between-group mean difference for each primary comparison, a) diagnosis and explanation without being sent for x-ray vs diagnosis and explanation based on x-ray reports (with showing patients their x-ray images) and b) diagnosis and explanation based on x-ray reports (without showing patients their x-ray images) vs diagnosis and explanation based on x-ray reports (with showing patients their x-ray images). Results will be presented as estimated mean differences between groups, with two-sided 95% confidence intervals and p-values (multiplicity adjusted for the two primary outcomes and two primary comparisons). Similar linear regression models will be used to compare the primary outcomes across the secondary comparison, diagnosis and explanation without being sent for x-ray vs diagnosis and explanation based on x-ray reports (without showing patients their x-ray images), to estimate the between-group mean difference, with two-sided 95% confidence intervals and p-values.

7.1.2 Secondary outcomes

All secondary outcomes will be compared across the two primary comparisons and the secondary comparison. Separate linear regression models for each continuous secondary outcome post-intervention will be used to estimate the between-group mean differences, with two-sided 95% confidence intervals and p-values.

Standard diagnostic plots will be used to check model assumptions, including assessing linearity and homoscedasticity, where appropriate. All analysis models will be adjusted for the stratification factor knee pain in the past 3 months (yes/no).

7.1.3 Subgroup analyses

We will assess whether the effect of a) diagnosis and explanation without being sent for x-ray compared to diagnosis and explanation based on x-ray reports (with showing patients their x-ray images) and b) diagnosis and explanation based on x-ray reports (without showing patients their x-ray images) compared to diagnosis and explanation based on x-ray reports (with showing patients their x-ray images) on each of the primary outcomes, i) belief about joint replacement surgery and ii) belief about exercise and physical activity, is moderated by knee pain in the past 3 months (yes/no). Separate linear regression models will be fitted for each primary outcome, with treatment group, knee pain in past 3 months, and an interaction between treatment group and knee pain in the past 3 months.

7.2 Measures to Adjust for Multiplicity, Confounders, Heterogeneity

We have two primary outcomes across two primary comparisons. We have used a conservative Bonferroni correction to account for these two primary outcomes and two primary pair-wise comparisons, and adjusted the alpha to 0.0125 to maintain an overall alpha of 0.05 across the two primary outcomes and comparisons. We have several secondary outcomes. All secondary outcomes are exploratory. We will therefore not adjust for multiple secondary outcomes but instead report all effect sizes, confidence intervals, and p values in order to let readers use their own judgment about the relative weight of the conclusions. This approach aligns with the usage of p-values favoured by the American Statistical Association [7].

8 Sensitivity Analyses

No sensitivity analyses will be performed.

9 QC Plans

Data quality will be checked/promoted through a process of identifying extreme values and checking the source of these values in case of a data entry error. A record of any/all manual corrections to data will be maintained. Calculations of scores from multi-item scales will be carried out using Microsoft Excel functions and cross-checked using other statistical packages to reduce errors.

10 Programming Plans

A list of all tables, figures, listings and their templates can be found in Appendix 3.

11 References

1. Lawford, B., et al., *Effect of information content and general practitioner recommendation on treatment beliefs and intentions for knee osteoarthritis: An online multi-arm randomised controlled trial.* ACR Open Rheumatology, 2023. **5**(1): p. 17-27.

2. Shelby, R.A., et al., *Brief Fear of Movement Scale for osteoarthritis.* Arthritis Care & Research, 2012. **64**(6): p. 862-71.

3. Carpenter, J. and M. Kenward, *Multiple imputation and its application*. 2013, West Sussex, UK: John Wiley & Sons, Ltd.

4. Haber, T., et al., *Effects of Hip Pain Diagnostic Labels and Their Explanations on Beliefs About Hip Pain and How to Manage It: An Online Randomized Controlled Trial.* Journal of Orthopaedic & Sports Physical Therapy, 2023. **53**(11): p. 673-684.

5. Lawford, B.J., et al., *Removing Pathoanatomical Content From Information Pamphlets About Knee Osteoarthritis Did Not Affect Beliefs About Imaging or Surgery, but Led to Lower Perceptions That Exercise Is Damaging and Better Osteoarthritis Knowledge: An Online Randomised Controlled Trial.* J Orthop Sports Phys Ther, 2023. **53**(4): p. 1-15.

6. Wasserstein, R.L., A.L. Schirm, and N.A. Lazar, *Moving to a world beyond “p< 0.05”*. 2019, Taylor & Francis.
