# Supplementary material for: Effects of X-ray–based diagnosis and explanation of knee osteoarthritis on patient beliefs about osteoarthritis management: A randomised clinical trial
Source: PLoS Med. 2025 Feb 4;22(2):e1004537. doi: 10.1371/journal.pmed.1004537 (PMC11838874; doi:10.1371/journal.pmed.1004537)
Supplement: S4 Appendix — (DOCX) [file pmed.1004537.s004.docx]

# S4 Appendix. Baseline descriptive demographic measures

| **Domain** | **Question** | **Scale** |
| --- | --- | --- |
| Gender | *Are you…* | Male  Female  Transgender male  Transgender female  Gender variant/non-conforming  Prefer not to say |
| Age | *What is your age?* | Self-reported in years |
| Ethnicity | *With what ethnicity do you most identify?* | Australian/New Zealand  Aboriginal and/or Torres Strait Islander  European  Asian  Other Oceania  North African & Middle Eastern  Sub-Saharan Africa  North American  South American  Prefer not to say  Other (please specify) |
| State living in | *What state do you live in?* | ACT  NSW  NT  QLD  SA  TAS  VIC  WA |
| Height | *What is your height?* | Self-reported in metres |
| Weight | *What is your weight?* | Self-reported in kilograms |
| Level of education | *What is the highest level of education you have completed?* | Primary school  High school  Trade or trade certificate  University or tertiary institute degree  Higher university degree (e.g. Masters, PhD)  Don’t know/unsure |
| Financial situation | *How would you describe your financial situation?* | Find it a strain to get by from week to week  Have to be careful with money  Able to manage without much difficulty  Quite comfortably off  Very comfortably off  Prefer not to answer |
| Level of exercise | *Do you currently participate in any type of regular exercise and/or physical activity (e.g. strengthening program, tennis, walking, cycling etc)* | No  Yes, 0-1 times per week  Yes, 2-3 times per week  Yes, 4-5 times per week  Yes, 6+ times per week |
| Medication use | *Do you regularly take pain relief medication for a musculoskeletal (i.e., bone/muscle/joint) condition?* | Yes  No |
| X-ray history | *Have you ever had an x-ray before?* | Yes  No  Not sure |
| Level of literacy for health information | *How easily can you read and understand written health information? I find it…* | 5-point Likert scale with response options:  Very difficult  Difficult  Neither easy nor difficult  Easy  Very easy |
| Painful joint | In which knee joint(s) have you experienced pain in the past 3 months? | Left knee only  Right knee only  Both knees |
| Pain | Select the number which indicates the average amount of pain felt over the PAST WEEK in your left knee/right knee/both knees. | NRS ranging from 0 (‘no pain’) to 10 (‘worst pain possible’) |
| Physical function | Select the number which indicates how much your left knee /right knee/both knees have interfered with your physical function over the PAST WEEK. | NRS ranging from 0 (‘no interference’) to 10 (‘maximal interference with function’) |
