# Supplementary material for: Effects of X-ray–based diagnosis and explanation of knee osteoarthritis on patient beliefs about osteoarthritis management: A randomised clinical trial
Source: PLoS Med. 2025 Feb 4;22(2):e1004537. doi: 10.1371/journal.pmed.1004537 (PMC11838874; doi:10.1371/journal.pmed.1004537)
Supplement: S5 Appendix — (DOCX) [file pmed.1004537.s005.docx]

# S5 Appendix. Hypothetical scenario participants were asked to consider before being randomised to a group.

Imagine that one of your knees has been painful for about six months. At first, the pain was on-and-off, but lately has been getting worse and it is now bothering you on most days. You feel the knee pain during walking and using the stairs, but are unsure what caused the pain to start. Imagine that you have made an appointment with a general practitioner (GP) to find out what is wrong with your knee.
