# Supplementary material for: Effects of X-ray–based diagnosis and explanation of knee osteoarthritis on patient beliefs about osteoarthritis management: A randomised clinical trial
Source: PLoS Med. 2025 Feb 4;22(2):e1004537. doi: 10.1371/journal.pmed.1004537 (PMC11838874; doi:10.1371/journal.pmed.1004537)
Supplement: S6 Appendix — (DOCX) [file pmed.1004537.s006.docx]

# S6 Appendix. Primary and secondary outcomes and process measures

| **Domain** | **Question** | **Scale** |
| --- | --- | --- |
| **Primary outcome measures** | | |
| Belief about joint replacement surgery | *Based on the video you have just watched, do you think joint replacement surgery (to replace the affected joint with an artificial joint) would be necessary for your hypothetical knee osteoarthritis at some stage?* | 11-point NRS ranging from 0=definitely not necessary to 10=definitely necessary |
| Belief about exercise and physical activity | *Based on the video you have just watched, do you think exercise and physical activity would be helpful to manage your hypothetical knee osteoarthritis?* | 11-point NRS ranging from 0=definitely not helpful to 10=definitely helpful |
| **Secondary outcome measures** | | |
| **Beliefs about treatment options** | | |
| Belief about safety of exercise | *Based on the video you have just watched, do you think exercise and physical activity could damage your hypothetical knee osteoarthritis?* | 11-point NRS ranging from 0=definitely would not damage it to 10=definitely would damage it |
| Belief about medication | *Based on the video you have just watched, do you think medication would help you manage your hypothetical knee osteoarthritis?* | 11-point NRS ranging from 0=definitely not helpful to 10=definitely helpful |
| **Level of concern** | | |
| Level of concern | *Based on the video you have just watched, how concerned would you be that your hypothetical knee osteoarthritis would get worse in the future?* | 11-point NRS ranging from 0=not concerned to 10=very concerned |
| **Fear of movement** | | |
| Brief Fear of Movement Scale ([32](#_ENREF_32)) | *Based on the video you have just watched, and thinking about your hypothetical knee osteoarthritis, please answer the following questions:*   1. *I'm afraid that I might injure myself if I exercise* 2. *If I were to try to overcome it, my pain would increase* 3. *I am afraid that I might injure myself accidentally* 4. *Simply being careful that I do not make any unnecessary movements is the safest thing I can do to prevent my pain from worsening* 5. *It's really not safe for a person with a condition like mine to be physically active* 6. *I can't do all the things normal people do because it's too easy for me to get injured* | 4-point scale ranging “strongly disagree” (1), “disagree” (2), “agree” (3), and “strongly agree” (4)  Each item is scored 1-4. Scores are summed for an overall score ranging 6-24. Higher scores indicate greater fear of movement. |
| **Perceptions about healthcare providers** | | |
| Belief about orthopaedic surgeon | *Based on the video you have just watched, how much do you think an orthopaedic surgeon could help you with your hypothetical knee osteoarthritis?* | 11-point NRS ranging from 0=definitely could not help to 10=definitely could help |
| Belief about rheumatologist | *Based on the video you have just watched, how much do you think a rheumatologist could help you with your hypothetical knee osteoarthritis?* | 11-point NRS ranging from 0=definitely could not help to 10=definitely could help |
| Belief about physiotherapist | *Based on the video you have just watched, how much do you think a physiotherapist could help you with your hypothetical knee osteoarthritis?* | 11-point NRS ranging from 0=definitely could not help to 10=definitely could help |
| **Satisfaction** | | |
| Overall satisfaction | *Based on the video you have just watched, how satisfied would you be with this initial GP consultation about your hypothetical knee osteoarthritis?* | 11-point NRS ranging from 0=definitely not satisfied to 10=definitely satisfied |
| Satisfaction with information | *Based on the video you have just watched, how satisfied would you be with the information you received from the GP about your hypothetical knee osteoarthritis?* | 11-point NRS ranging from 0=definitely not satisfied to 10=definitely satisfied |
| Confidence | *Based on the video you have just watched, how confident would you be that the GP has provided an accurate diagnosis of your hypothetical knee osteoarthritis?* | 11-point NRS ranging from 0=not at all confident to 10=very confident |
| **Process measures** | | |
| Whether watched allocated video or not | *Did you watch the video all the way through?* | Yes  No |
| Time spent watching video | Recorded by Qualtrics (time spent on page) | In minutes |
