# Supplementary material for: Effects of X-ray–based diagnosis and explanation of knee osteoarthritis on patient beliefs about osteoarthritis management: A randomised clinical trial
Source: PLoS Med. 2025 Feb 4;22(2):e1004537. doi: 10.1371/journal.pmed.1004537 (PMC11838874; doi:10.1371/journal.pmed.1004537)
Supplement: S7 Appendix — (DOCX) [file pmed.1004537.s007.docx]

# S7 Appendix. Baseline characteristics of participants who did and did not complete at least one primary outcome, reported as mean (standard deviation) unless otherwise stated.

|  | **Incomplete at least one primary outcomes** | **Completed both primary outcomes** |
| --- | --- | --- |
|  | **[N=5]** | **[N=612]** |
| Age (years), median (IQR) | 59.0 (55.0-59.0) | 60.0 (50.0-68.0) |
| Gender, n (%) |  |  |
| Male | 1 (20%) | 274 (44.8%) |
| Female | 4 (80%) | 337 (55.1%) |
| Transgender male | 0 (0%) | 1 (0.2%) |
| Ethnicity, n (%) |  |  |
| Australian/New Zealand | 4 (80%) | 426 (70%) |
| Aboriginal and Torres Strait Islander | 0 (0%) | 9 (2%) |
| European | 0 (0%) | 111 (18%) |
| Asian | 0 (0%) | 37 (6%) |
| Other Oceania | 0 (0%) | 2 (<1%) |
| North African & Middle Eastern | 0 (0%) | 6 (1%) |
| Sub-Saharan Africa | 0 (0%) | 2 (<1%) |
| North American | 0 (0%) | 6 (1%) |
| South American | 0 (0%) | 3 (1%) |
| Other | 1 (20%) | 8 (1%) |
| Prefer not to answer | 0 (0%) | 2 (<1%) |
| State/territory, n (%) |  |  |
| Australian Capital Territory | 0 (0%) | 8 (1%) |
| New South Wales | 0 (0%) | 172 (28%) |
| Northern Territory | 0 (0%) | 4 (1%) |
| Queensland | 2 (40%) | 137 (22%) |
| South Australia | 0 (0%) | 54 (9%) |
| Tasmania | 1 (20%) | 14 (2%) |
| Victoria | 1 (20%) | 173 (28%) |
| Western Australia | 1 (20%) | 50 (8%) |
| Height (m) | 1.6 (0.1) | 1.7 (0.1) |
| Weight (kg) | 74.8 (17.8) | 79.2 (19.1) |
| Body mass index (kg/m^2^) | 27.5 (5.9) | 28.2 (7.3) |
| Highest education level, n (%) |  |  |
| Primary school | 0 (0%) | 9 (2%) |
| Secondary school | 2 (40%) | 202 (33%) |
| Trade or trade certificate | 0 (0%) | 146 (24%) |
| University or tertiary institute degree | 2 (40%) | 203 (33%) |
| Higher university degree (e.g. Masters, PhD) | 1 (20%) | 51 (8%) |
| Don't know/unsure | 0 (0%) | 1 (<1%) |
| Financial situation, n (%) |  |  |
| Find it a strain to get by from week to week | 0 (0%) | 87 (14%) |
| Have to be careful with money | 0 (0%) | 251 (41%) |
| Able to manage without much difficulty | 1 (20%) | 159 (26%) |
| Quite comfortably off | 3 (60%) | 83 (14%) |
| Very comfortably off | 1 (20%) | 28 (5%) |
| Prefer not to answer | 0 (0%) | 4 (1%) |
| Participation in regular exercise/physical activity, n (%) |  |  |
| None | 1 (20%) | 177 (29%) |
| Yes, 1 time per week | 0 (0%) | 67 (11%) |
| Yes, 2-3 times per week | 0 (0%) | 178 (29%) |
| Yes, 4-5 times per week | 3 (60%) | 121 (20%) |
| Yes, 6+ times per week | 1 (20%) | 69 (11%) |
| Activity-related knee pain in the last 3 months, n (%) | 0 (0%) | 307 (50%) |
| Joint with knee pain^*^, n (%) |  |  |
| Left knee only | 0 (0%) | 90 (29%) |
| Right knee only | 0 (0%) | 103 (34%) |
| Both knees | 0 (0%) | 114 (37%) |
| Self-reported knee pain (NRS) | .. | 4.5 (2.1) |
| Self-reported physical function (NRS) | .. | 4.1 (2.5) |
| Regular pain relief for musculoskeletal condition, n (%) | 1 (20%) | 184 (30%) |
| Ability to read and understand written health information^#^, median (IQR) | 5.0 (5.0-5.0) | 4.0 (4.0-5.0) |

IQR: interquartile range (25^th^ to 75^th^ percentile); kg: kilograms; m: metres; NRS: numerical rating scale ranging from 0 (‘no pain’ or ‘no interference’) to 10 (‘worst pain possible’ or ‘maximal interference with function’).

*The denominator for joint with knee pain was the number of participants with knee pain in each group.

^#^Rated using a 5-point scale with terminal descriptors of 1=‘always difficult’ to 5=‘always easy’.
