# Supplementary material for: Effects of X-ray–based diagnosis and explanation of knee osteoarthritis on patient beliefs about osteoarthritis management: A randomised clinical trial
Source: PLoS Med. 2025 Feb 4;22(2):e1004537. doi: 10.1371/journal.pmed.1004537 (PMC11838874; doi:10.1371/journal.pmed.1004537)
Supplement: S8 Appendix — (DOCX) [file pmed.1004537.s008.docx]

# S8 Appendix: Moderation of intervention effect by lived experience of knee pain on primary outcomes using complete case data.

| **Potential moderators at baseline** | | **Group 1**  **Mean (SD)** | **Group 2**  **Mean (SD)** | | | **Group 3**  **Mean (SD)** | **Primary comparison 1**  **Group 3 vs 1** | | **Primary comparison 2**  **Group 3 vs 2** | |
| --- | --- | --- | --- | --- | --- | --- | --- | --- | --- | --- |
|  |  | **Clinical explanation (no x-rays) [N=208]^a^** | **Radiographic explanation (not showing x-ray images) [N=203]^a^** | | | **Radiographic explanation (showing x-ray images)**  **[N=206]^a^** | **Mean**  **difference^b^**  **(95% CI)** | **Interaction p-value** | **Mean difference^b^ (95% CI)** | **Interaction p-value** |
|  | **Belief joint replacement surgery is necessary at some stage*** | | | | | | | | | |
| Lived experiences with knee pain | |  | |  |  |  |  |  |  |  |
| Yes | | 3.5 (2.6) | 3.9 (2.7) | | | 4.2 (2.6) | 0.7 (-0.1, 1.4) | 0.094 | 0.3 (-0.4, 1.0) | 0.416 |
| No | | 3.2 (2.8) | 4.0 (2.4) | | | 4.7 (2.7) | 1.6 (0.8, 2.3) |  | 0.7 (0.0, 1.4) |  |
|  | **Belief exercise and physical activity is helpful^¥^** | | | | | | | | | |
| Lived experience with knee pain | |  | |  |  |  |  |  |  |  |
| Yes | | 7.8 (1.7) | 7.6 (1.9) | | | 7.2 (2.3) | -0.6 (-1.1, 0.0) | 0.468 | -0.4 (-1.0, 0.2) | 0.329 |
| No | | 8.0 (2.0) | 7.7 (2.1) | | | 7.7 (2.1) | -0.3 (-0.8, 0.3) |  | 0.0 (-0.6, 0.6) |  |

CI=confidence intervals; SD=standard deviation.

Positive between-group differences indicate higher score in: Group 3 (for Group 3 vs 1 and Group 3 vs 2).

^a^5 participants missing all primary and secondary outcomes (3 in Group 1, 1 in Group 2, and 1 in Group 3).

^b^Difference between groups was obtained by fitting separate linear regression models for each primary outcome with an interaction term between the intervention group and lived experience with knee pain.

*Measured using the 11-point numerical rating scale ranging from 0=’definitely not necessary’ to 10=’definitely necessary’.

^¥^Measured using the 11-point numerical rating scale ranging from 0=’definitely not helpful’ to 10=’definitely helpful’.
