# Supplementary material for: Effects of X-ray–based diagnosis and explanation of knee osteoarthritis on patient beliefs about osteoarthritis management: A randomised clinical trial
Source: PLoS Med. 2025 Feb 4;22(2):e1004537. doi: 10.1371/journal.pmed.1004537 (PMC11838874; doi:10.1371/journal.pmed.1004537)
Supplement: S9 Appendix — (DOCX) [file pmed.1004537.s009.docx]

# S9 Appendix. Process measures.

|  | **Clinical explanation (no x-rays)** | **Radiographic explanation (not showing x-ray images)^*^** | **Radiographic explanation (showing x-ray images)** |
| --- | --- | --- | --- |
|  | **[N=208]** | **[N=203]** | **[N=206]** |
| Video and audio quality was sufficient, n (%) | 205 (99%) | 202 (100%) | 203 (99%) |
| Time spent watching video (minutes), median (IQR) | 2.1 (2.0-2.3) | 2.2 (2.1-2.4) | 2.3 (2.2-2.5) |

IQR = interquartile range (25th to 75th percentile).

*1 participant missing. The denominator included 202 participants with available data.
