# Supplementary material for: Effects of X-ray–based diagnosis and explanation of knee osteoarthritis on patient beliefs about osteoarthritis management: A randomised clinical trial
Source: PLoS Med. 2025 Feb 4;22(2):e1004537. doi: 10.1371/journal.pmed.1004537 (PMC11838874; doi:10.1371/journal.pmed.1004537)
Supplement: S10 Appendix — (DOCX) [file pmed.1004537.s010.docx]

# S10 Appendix. X-ray images shown in the *radiographic explanation (showing x-rays)* group


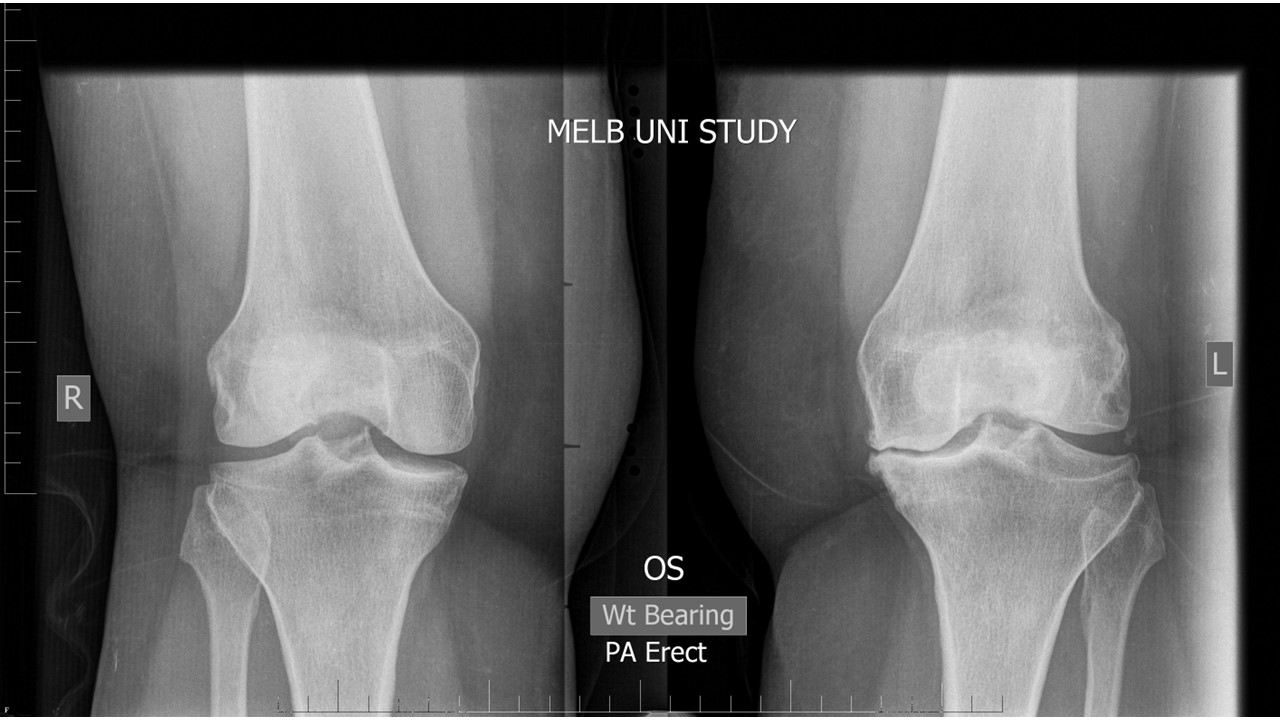


**IMAGE A**


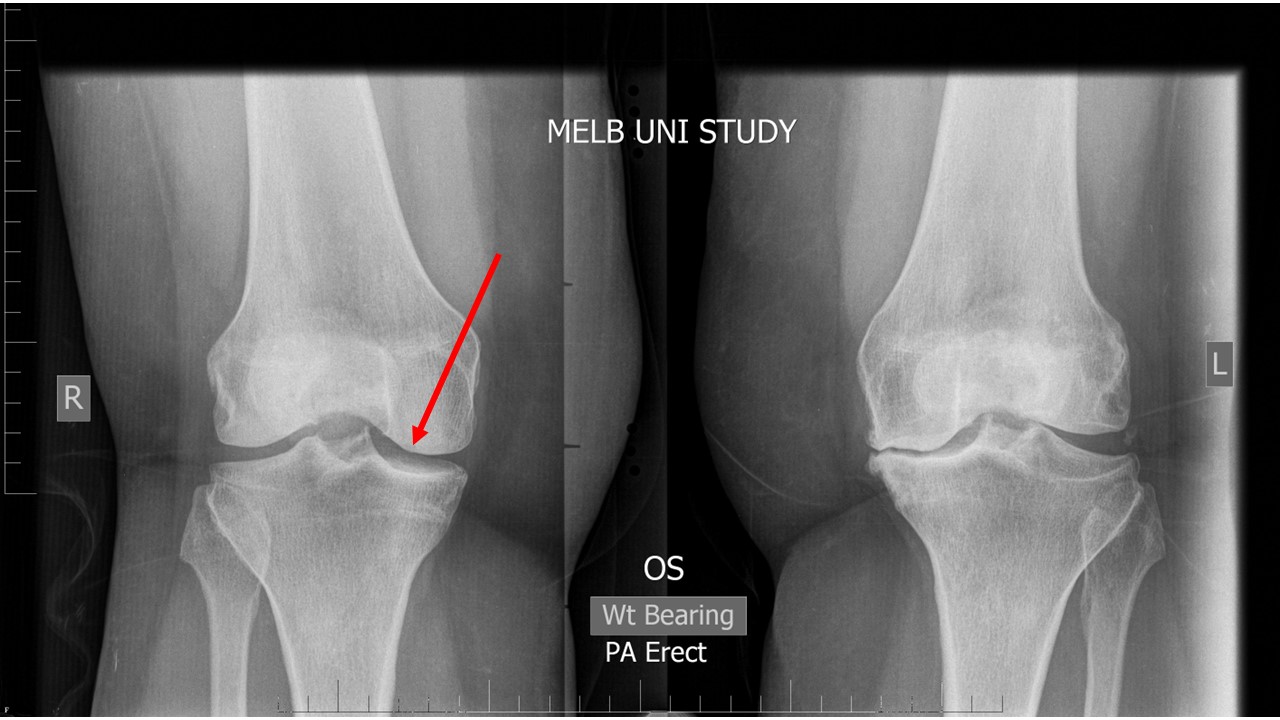


**IMAGE B**


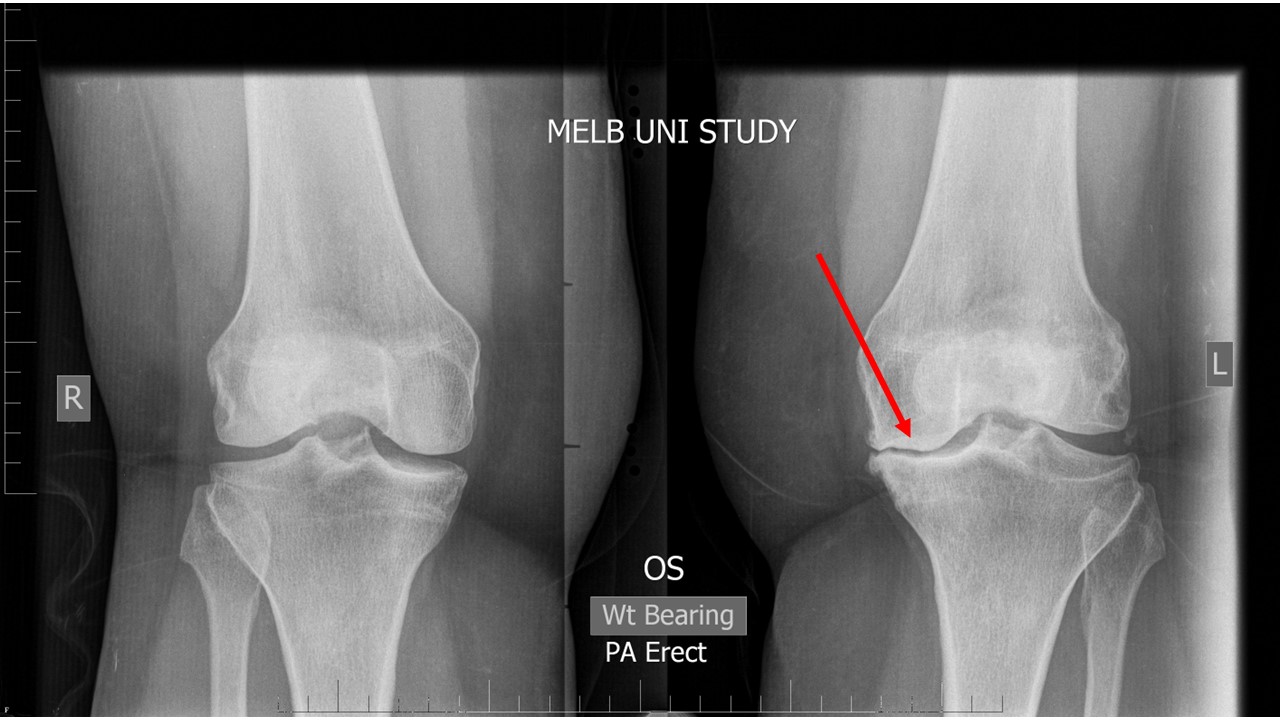


**IMAGE C**

**
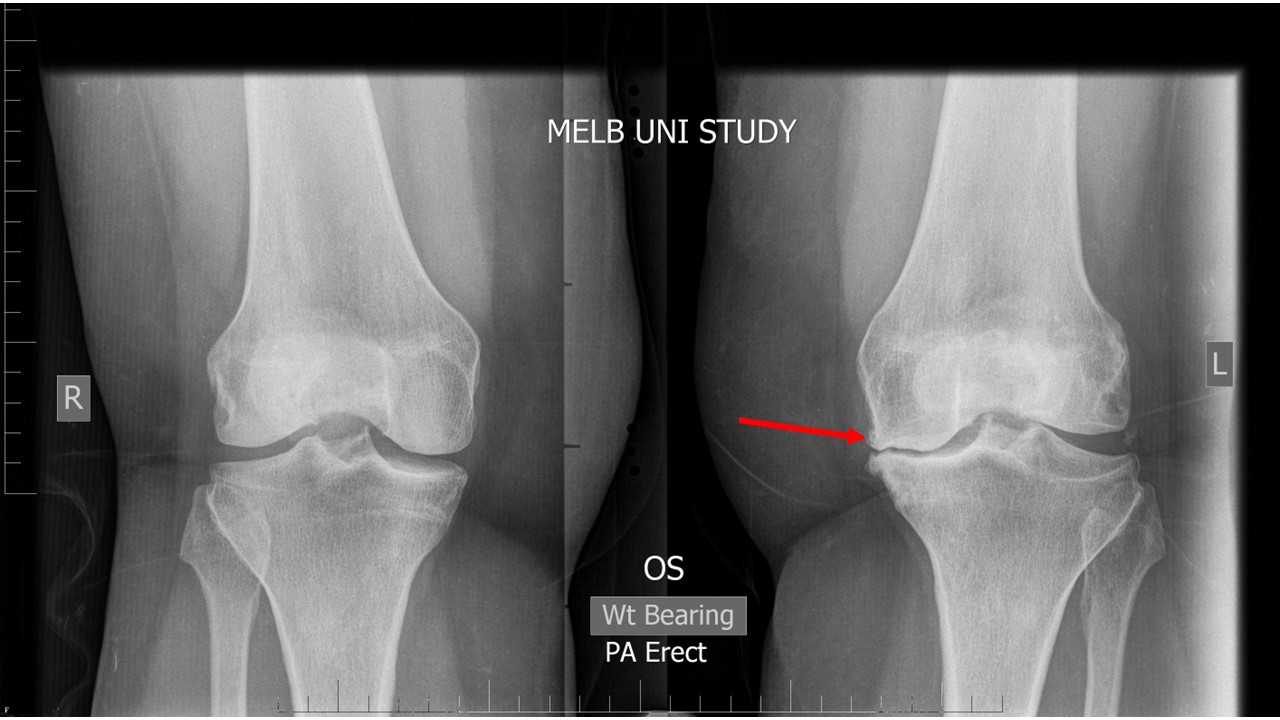
**

**IMAGE D**
