# Supplementary material for: Effects of X-ray–based diagnosis and explanation of knee osteoarthritis on patient beliefs about osteoarthritis management: A randomised clinical trial
Source: PLoS Med. 2025 Feb 4;22(2):e1004537. doi: 10.1371/journal.pmed.1004537 (PMC11838874; doi:10.1371/journal.pmed.1004537)
Supplement: Attachment — Submitted filename: PLOS Medicine response to reviewer comments_v1.docx [file pmed.1004537.s015.docx]

19th December 2024

Dear Dr Van Epps,

Re: PMEDICINE-D-24-03202R1 Manuscript revisions

Thank you very much for the opportunity to revise our manuscript for publication in *PLOS Medicine.* In the following pages, we have outlined our responses to the comments of the reviewers. We also describe the changes we have made to the manuscript.

We hope that you find our revised manuscript satisfactory.

Yours sincerely,

Belinda Lawford

On behalf of the authors

**Comments from the editors/academic editor:**

**1.      We felt that the discussion could be expanded to further contextualize the findings relative to real-world clinical scenarios; for example, the scenario of GPs providing no clinical explanation to patients with knee OA (ie, radiographic explanation only) is not well-documented, and it would be useful cite any previous studies that address this (current citations focus more on prescription trends for imaging).**

Author action: We have expanded the discussion as suggested:

Line 441, page 24: “*Implications for clinical practice (Appendix 8)*

*Our findings provide empirical evidence of the potential undesirable effects of using x-rays to diagnose and explain OA.* Clinical guidelines *and care standards* advocate against routine use of imaging for diagnosing OA because there is no evidence it is beneficial for diagnosis [6, 18, 47], *instead* recommend*ing* that healthcare providers diagnose knee OA clinically whenever possible. *However, trends in use of imaging for knee OA in Australia, the UK, and US have remained largely unchanged over time [**8**,* *19**,* *47**]. In the UK, knee radiographs have been ranked as one of the most over-used tests in primary care [**49**]. In the US, x-rays are used for diagnosis of OA in 87% of patients presenting to primary care [**19**] and in Australia, 46% and 56% of patients presenting to a general practitioner for management of knee or hip OA respectively are referred for imaging [**8**]. It is thus not surprising that qualitative research in Australia has shown that most general practitioners prefer to use imaging to confirm a knee OA diagnosis, and that they describe OA to their patients as a problem of cartilage degeneration, joint space narrowing on x-ray, or “wear and tear” [**22**]. When general practitioners do provide a diagnosis of OA, they often avoid the term OA in favour of “wear and tear” [**50**] or use terms taken directly from radiology reports [**51**], which can convey a range of negative meanings to patients [**52**].*

*General practitioners may decide to order imaging for numerous reasons, including lack of time to discuss the risks and potential harms of imaging versus benefits, concern about malpractice, a desire to meet patient expectations, perceived need to exclude other diagnoses, or lack of confidence to diagnose OA without x-ray [**22**,* *48**].* Changing clinical practice will require a substantial shift in long-held beliefs and habits [22] and our findings are a first step by showing that *use of x-rays to diagnose and explain knee OA* causally impacts patient beliefs *about OA management*. If x-ray is necessary, strategies to reduce potentially undesirable effects on patient beliefs may include reassuring patients that pathoanatomical changes *do not necessarily predict prognosis* and adapting the language and terminology used in x-ray reports (e.g., including epidemiological information about age-relevant prevalence of imaging characteristics in asymptomatic individuals) [41, 53, 54].”

**2.      The current data availability statement does not comply with the PLOS Data Availability Policy (see** [**http://journals.plos.org/plosmedicine/s/data-availability)**](http://journals.plos.org/plosmedicine/s/data-availability))**, which requires that all data underlying the study's findings be provided in a repository or as Supporting Information. For data residing with a third party, authors are required to provide instructions with contact information (web or email address) for obtaining the data. Please note that a study author cannot be the contact person for the data. PLOS journals do not allow statements supported by "data not shown" or "unpublished results." For such statements, authors must provide supporting data or cite public sources that include it.**

Author action: We have deposited the data on a repository as required and updated the data availability statement accordingly:

“**Data sharing**: *Data are available on Dryad Digital Repository (**56**).*”

**3.      Abstract: please include details of the planned statistical analyses (primary and secondary comparisons).**

Author action: We have updated the abstract as suggested:

***Methods and findings.*** This was a 3-arm randomised controlled trial conducted in May 2024 as a single exposure (no follow-up) online survey. 617 people aged ≥45 years, with and without chronic knee pain, were recruited from the Australian-wide community. Participants considered a hypothetical scenario where their knee was painful for six-months and they had made an appointment with a general practitioner (primary care physician). Participants were randomly allocated to one of three groups where they watched a 2-minute video of the general practitioner providing them with either: i) *clinical* *explanation of knee osteoarthritis (no x-rays)*; ii) *radiographic explanation (not showing x-ray images)* or; iii) *radiographic explanation (showing x-ray images)*. *Primary comparisons were: 1) clinical explanation (no x-rays) versus radiographic explanation (showing x-ray images), and; 2) radiographic explanation (not showing x-ray images) versus radiographic explanation (showing x-ray images). The secondary comparison was clinical explanation (no x-rays) versus radiographic explanation (not showing x-ray images).* Primary outcomes were perceived i) necessity of joint replacement surgery; and ii) helpfulness of exercise and physical activity, measured on 11-point numeric rating scales (NRS) ranging 0-10.

**4.      Please include the full recruitment dates (Month, Day, Year) in the Abstract, with beginning and ending dates.**

Author action: Full recruitment dates have been added as requested:

***Methods and findings***. This was a 3-arm randomised controlled trial conducted *between May 23 2024 and May 28 2024* as a single exposure (no follow-up) online survey. 617 people aged ≥45 years, with and without chronic knee pain, were recruited from the Australian-wide community.

**5.      We take an all-or-none approach to inclusion of secondary outcomes in the abstract. If you choose to include secondary outcomes, they must all be included (methods and findings) to avoid selective reporting. Given the large number of secondary outcomes, we prefer that the abstract be limited to the primary outcome (per CONSORT).**

Author action: As suggested, we have now removed reporting of secondary outcomes from the abstract.

**6.      Methods: please check the URL provided for the SAP, as the link does not appear to be correct/functional.**

Author action: Apologies, the link (page 9) was not correctly hyperlinked, but should be functional now.

**7.      Please consider moving Figure 2 to the supplement, as we were not convinced that this graphic adds to the numerical reporting of the outcomes (although it would be very useful for oral presentations).**

Author action: This has been moved to Appendix 8 as suggested.

**8.      When you submit your revision, please include the original protocol (uploaded as a supplemental file) and the SAP (if a separate document). It was an oversight that these documents were not requested prior to peer review.**

Author action: These have been added to our supplementary material as requested.

**Comments from the reviewers:**

**Reviewer #1: Statistical review**

**This paper reports a three-arm RCT comparing hypothetical consultation strategies for individuals with knee osteoarthritis. I found the study design to be an interesting approach to investigate beliefs. The statistical methods are suitable and are mostly well-reported. I had only some minor comments, provided below:**

**1.      Abstract: it would be useful to clarify that the first outcome is also a 11 point NRS - at the moment it is not clear that this is the case and it could be that it's a binary outcome. Perhaps add 'both' before 'measured on' on line 51.**

Author action: We have edited this sentence as suggested:

Under ‘Methods and findings’ section of the abstract: “Primary outcomes were perceived i) necessity of joint replacement surgery; and ii) helpfulness of exercise and physical activity, *both* measured on 11-point numeric rating scales (NRS) ranging 0-10.”

**2.      Abstract: I would recommend providing the mean NRS per group as well as the mean difference + confidence interval. I would also provide this for the exercise/physical activity outcome.**

Author action: We have added this data as suggested:

Under ‘Methods and findings’ section of the abstract: “Compared to *clinical explanation (no x-rays)*, those who received *radiographic explanation (showing x-ray images)* believed surgery was more necessary (*mean 3.3 [standard deviation: 2.7] versus 4.5 [2.7], respectively;* mean difference 1.1 [95% confidence interval: 0.5, 1.8]), but there were no differences in beliefs about the helpfulness of exercise and physical activity (*mean 7.9 [standard deviation: 1.9] versus 7.5 [2.2], respectively; mean difference -0.4 [95% confidence interval: -0.9, 0.1]*). There were no differences in beliefs between *radiographic explanation* with and without showing x-ray images *(for beliefs about necessity of surgery: mean 4.5 [standard deviation: 2.7] versus 3.9 [2.6] respectively; mean difference 0.5 [95% confidence interval: -0.1, 1.2]; for beliefs about helpfulness of exercise and physical activity: mean 7.5 [standard deviation: 2.2] versus 7.7 [2.0] respectively; mean difference -0.2 [95% confidence interval: -0.7, 0.3])*.”

**3.      Abstract: given the number of secondary outcomes in the trial registration page, I would recommend either not reporting results of secondary outcomes in the abstract, or briefly mentioning them all e.g. 'no significant differences were found for other outcomes including…'.**

Author action: We have now removed secondary outcomes from the abstract as instructed by the editors.

**4.      Table 3: I would recommend reporting p-values to 2 significant figures or <0.001 (I think this is the PLOS guidance).**

Author action: We have now reported p-values to 3 significant figures, as instructed in the accompanying editorial requests.

**Reviewer #2**

**This is a really interesting study on the potential effect of radiographic diagnosis on patients' beliefs about the management of their knee OA. I commend the authors for undertaking a well-designed and well-reported study examining the differences between clinical and radiographic diagnoses  (without and with showing x-rays) using a hypothetical scenario. I have only a few minor queries about this work.**

Author response: We thank the reviewer for their comments and suggestions.

**1.      I wondered if the authors had any information or indication on why people failed to complete the survey, as this was quite a large proportion? What implications could this have had on the findings of the trial?**

Author response: As described in Figure 1, people were automatically excluded when the survey quota was reached, which happened when we had obtained our desired sample. When this occurred, any people who were in the middle of the survey (but had not yet been randomised) had their survey automatically ended. Given the rapid recruitment rate (all participants were recruited within five days) this meant that a large number of participants were subsequently excluded via this method. We do not believe that this had any implications on the findings of the trial.

Author action: We have made some minor edits to Figure 1 to state more clearly why these participants were excluded:

“Excluded (n=755)

- Did not read plain language statement (n=15)
- Did not consent to participate (n=37)
- Aged <45 years (n=28)
- Have consulted with a healthcare provider for chronic knee pain before (n=523)
- Discontinued survey (*includes those who had their survey automatically ended* once *the sample size* quota *was* reached) before randomisation (n=152)”

**2.      Previous research in electronic health records databases has shown that lots of patients with knee pain don't get a diagnosis of knee OA at their first consultation for their knee pain/knee problem. In the trial, patients who had previously consulted for knee pain were understandably excluded from this trial; however, their experience may also be important to consider, and I feel this should be acknowledged.**

Author action: We have added to the limitations section of our discussion to acknowledge this point:

Line 477 page 26: “Participants with and without knee pain were asked to respond to a hypothetical scenario. While we found no moderating effect of the lived experience of recent knee pain on outcomes, *our findings* may not necessarily translate to real-world clinical situations. Although many outcomes met or exceeded our *a priori* minimal 1.0-unit difference immediately after watching the videos, *it is unclear* whether these effects *on patient beliefs would impact their subsequent OA management behaviours*. As we included only people who spoke and read English, and *those who had never previously consulted a clinician for chronic knee pain, our findings may not be generalisable to non-English speaking people or those who have sought care for chronic knee pain.*”

**3.      Can the authors comment on the impact of negative social media and negative messages that still exist in society, such as 'wear and tear', and the role they may have played on the study participants' beliefs and the expectations that a joint replacement will be needed, in general, but also with reference to the those who received a radiographic diagnosis?**

Author action: We have added to our discussion to acknowledge how beliefs and perceptions may have been influenced by the impact of consumer information:

Line 395, page 22: “Our work provides the first causal evidence that using x-rays rather than a clinical approach to diagnose and explain knee OA can have potentially undesirable impacts on patient beliefs about knee OA. Prior qualitative research found that people with OA who receive x-rays of their joint believe that they need to protect it from further damage [21, 24, 36]. Our work also agrees with prior research showing that people who are given pathoanatomical OA educational information have unfavourable beliefs about OA and its management [15, 28, 29, 33, 37]. *For participants in our study, receiving an x-ray based diagnosis and explanation of knee OA may have reinforced existing misconceptions about OA, given the prevailing pathoanatomical discourse in current consumer OA information, which often employs biomedical terminology (e.g., ‘cartilage degeneration’, ‘bony spurs’) and visual representations that depict the joint as ‘damaged’ [**30**,* *38-41**].*”

**Reviewer #3:**

**This is a very interesting paper. In my opinion this is a well-conducted and thorough study. I have only minor points.**

Author response: We thank the reviewer for their comments and suggestions.

**1.      Abstract: Clearly written abstract, this highlights the study's main results. It probably won't fit to add the nuance of the third group.**

Author response: We thank the reviewer for their comment.

**2.      Methods: As I understand correctly you used a company to recruit people. Do they get paid for filling in a survey?**

Author action: We have added to our methods section to clarify that participants get paid a small incentive by the company we used to recruit people (Cint Pty Ltd):

Line 161, page 8: “Participants were recruited from across Australia (in any State or Territory) via a consumer network of people who self-nominated to participate in digital survey-based research (Cint Pty Ltd). *Participants receive a small financial incentive (approximately $2.55 AUD for completion of a 15- to 20-minute survey) from Cint to compensate them for their time and participation. Incentives were only provided after completing the entire survey to the end.*”

**3.      The link to the SAP is not working.**

Author action: Apologies, we have fixed the hyperlink on page 9 and it should now be working.

**4.      In the text I do not read if the GP is the same in each video, I guess so, but it is not mentioned.**

Author action: We have clarified that the GP is the same in each video:

Line 193, page 10: “Participants were then randomised to one of three groups where they watched a ~2-minute video of a GP (author DE, *who featured in all three videos*) providing them with a hypothetical: 1) *clinical explanation (no x-rays);* 2) *radiographic explanation (not showing x-ray images);* or 3) *radiographic explanation (showing x-ray images)*.”

**5.      Results: Figure 1 is clear. I was confused about the text saying 617 participants were enrolled and 523 were excluded. May be rephrase a bit.**

Author action: We have reworded this sentence for clarity:

Line 290, page 15: “*From the 1,372 people screened for eligibility*, we enrolled 617 participants between May 23 and May 28, 2024 (Figure 1). Major reasons for exclusion were previously having consulted a clinician for knee pain (n=523, 36%), discontinuing the survey before randomisation (n=152, 20%), or not consenting to participate (n=37, 5%). Characteristics of participants were similar between groups (Table 2). There were no differences between participants who did and did not complete primary outcomes (Appendix 4).”

**General editorial requests:**

**(Note: not all will apply to your paper, but please check each item carefully)

*       We ask every co-author listed on the manuscript to fill in a contributing author statement, making sure to declare all competing interests. If any of the co-authors have not filled in the statement, we will remind them to do so when the paper is revised. If all statements are not completed in a timely fashion this could hold up the re-review process. If new competing interests are declared later in the revision process, this may also hold up the submission. Should there be a problem getting one of your co-authors to fill in a statement we will be in contact. Please do not add or remove authors without first discussing this with the handling editor. You can see our competing interests policy here:** [**http://journals.plos.org/plosmedicine/s/competing-interests**](http://journals.plos.org/plosmedicine/s/competing-interests)**.**

Author response: Confirming that this has been completed.

***       Please upload any figures associated with your paper as individual TIF or EPS files with 300dpi resolution at resubmission; please read our figure guidelines for more information on our requirements:** [**http://journals.plos.org/plosmedicine/s/figures**](http://journals.plos.org/plosmedicine/s/figures)**. While revising your submission, please upload your figure files to the PACE digital diagnostic tool,** [**https://pacev2.apexcovantage.com/**](https://pacev2.apexcovantage.com/)**. PACE helps ensure that figures meet PLOS requirements. To use PACE, you must first register as a user. Then, login and navigate to the UPLOAD tab, where you will find detailed instructions on how to use the tool. If you encounter any issues or have any questions when using PACE, please email us at** [**PLOSMedicine@plos.org**](mailto:PLOSMedicine@plos.org)**.**

Author response: Confirming that this has been completed.

***       We expect all researchers with submissions to PLOS in which author-generated code underpins the findings in the manuscript to make all author-generated code available without restrictions upon publication of the work. In cases where code is central to the manuscript, we may require the code to be made available as a condition of publication. Authors are responsible for ensuring that the code is reusable and well documented. Please make any custom code available, either as part of your data deposition or as a supplementary file. Please add a sentence to your data availability statement regarding any code used in the study, e.g. "The code used in the analysis is available from Github [URL] and archived in Zenodo [DOI link]" Please review our guidelines at** [**https://journals.plos.org/plosmedicine/s/materials-software-and-code-sharing**](https://journals.plos.org/plosmedicine/s/materials-software-and-code-sharing) **and ensure that your code is shared in a way that follows best practice and facilitates reproducibility and reuse. Because Github depositions can
be readily changed or deleted, we encourage you to make a permanent DOI'd copy (e.g. in Zenodo) and provide the URL.**

Author response: No author-generated code was used in this study.

***       Please ensure that the study is reported according to the CONSORT guideline and include the completed CONSORT checklist (**[**https://www.equator-network.org/reporting-guidelines/consort/**](https://www.equator-network.org/reporting-guidelines/consort/)**) as Supporting Information. When completing the checklist, please use section and paragraph numbers, rather than page numbers. Please add the following statement, or similar, to the Methods: "This study is reported as per CONSORT guideline (S1 Checklist)."**

Author response: Confirming this has been completed.

**FORMATTING – GENERAL**

***       Abstract: Please structure your abstract using the PLOS Medicine headings (Background, Methods and Findings, Conclusions). Please combine the Methods and Findings sections into one section.**

Author response: Confirming this has been completed.

***       At this stage, we ask that you include a short, non-technical Author Summary of your research to make findings accessible to a wide audience that includes both scientists and non-scientists. The Author Summary should immediately follow the Abstract in your revised manuscript. This text is subject to editorial change and should be distinct from the scientific abstract. Ideally each sub-heading should contain 2-3 single sentence, concise bullet points containing the most salient points from your study. In the final bullet point of 'What Do These Findings Mean?', please include the main limitations of the study in non-technical language. Please see our author guidelines for more information:** [**https://journals.plos.org/plosmedicine/s/revising-your-manuscript#loc-author-summary**](https://journals.plos.org/plosmedicine/s/revising-your-manuscript#loc-author-summary)**.**

Author response: Confirming this has been added.

***       Please express the main results with 95% CIs as well as p values. When reporting p values please report as p<0.001 and where higher as the exact p value p=0.002, for example. Throughout, suggest reporting statistical information as follows to improve clarity for the reader "22% (95% CI [13%,28%]; p</=)". Please be sure to define all numerical values at first use.**

Author response: Confirming this has been reported.

***       Please include page numbers and line numbers in the manuscript file. Use continuous line numbers (do not restart the numbering on each page).**

Author response: Confirming this has been completed.

***       Please cite the reference numbers in square brackets. Citations should precede punctuation.**

Author response: Confirming this has been completed, as recommended on the submission guidelines website we have used Vancouver referencing style.

**FIGURES AND TABLES**

***       Please provide titles and legends for all figures and tables (including those in Supporting Information files).**

Author response: Confirming this has been completed.

***       Please define all abbreviations used in each figure/table (including those in Supporting Information files).**

Author response: Confirming this has been completed.

***       Please consider avoiding the use of red and green in order to make your figure more accessible to those with color blindness.**

Author response: Confirming this has been completed.

**SUPPLEMENTARY MATERIAL**

***       Please note that supplementary material will be posted as supplied by the authors. Therefore, please amend it according to the relevant comments outlined here.**

***       Please cite your Supporting Information as outlined here:** [**https://journals.plos.org/plosmedicine/s/supporting-information**](https://journals.plos.org/plosmedicine/s/supporting-information)

Author response: Confirming we have completed this.

**REFERENCES
*       PLOS uses the numbered citation (citation-sequence) method and first six authors, et al.**

Author response: Confirming we have completed this.

***       Please ensure that journal name abbreviations match those found in the National Center for Biotechnology Information (NCBI) databases (**[**http://www.ncbi.nlm.nih.gov/nlmcatalog/journals**](http://www.ncbi.nlm.nih.gov/nlmcatalog/journals)**), and are appropriately formatted and capitalised.**

Author response: Confirming we have completed this.

***       Where website addresses are cited, please include the complete URL and specify the date of access (e.g. [accessed: 12/06/2023]).**

Author response: Confirming we have completed this.

***       Please also see** [**https://journals.plos.org/plosmedicine/s/submission-guidelines#loc-references**](https://journals.plos.org/plosmedicine/s/submission-guidelines#loc-references) **for further details on reference formatting.**

**RCTs

*       PLOS Medicine requires that all trials be prospectively registered in one of registries recognized by WHO. Please ensure that study registration details are included in the Methods section.**

Author response: This has been included.

***       Please structure the Methods section using the following sub-headings: Study design and participants, Randomization and masking, Procedures, Outcomes, Statistical analysis.**

Author response: These subheadings have been added.

***       Please ensure that all prespecified outcomes (primary, secondary, and exploratory) are listed in the Methods/Outcomes section and indicate whether there are outcomes that are not presented in the current report.**

Author response: This has been completed.

***       Please specify the dates (Month Day, Year) during which study enrollment and follow up occurred.**

Author response: Confirming this has been completed.

***       Please include absolute numbers wherever you report percentages; eg, n/N (%)**

Author response: This has been completed.

***       Please present the safety data for the study including numbers of specific events and whether or not adverse events are thought to be related to treatment. AEs should be reported in the abstract, per CONSORT and CONSORT-Harms.**

Author response: N/A

***       Please ensure that all components of CONSORT are present in the manuscript, including how randomization was performed, allocation concealment, blinding of intervention, definition of lost to follow-up, power statement. As above, when completing the checklist, please use section and paragraph numbers, rather than page numbers.**

Author response: Confirming this has been completed.

***       Please report your abstract according to CONSORT for abstracts, following the PLOS Medicine abstract structure (Background, Methods and Findings, Conclusions)** [**https://www.equator-network.org/reporting-guidelines/consort-abstracts/**](https://www.equator-network.org/reporting-guidelines/consort-abstracts/)

Author response: Confirming this has been completed.

***       In keeping with our commitment to Open Science, please include the study protocol document and analysis plan (including any amendments) as Supporting Information to be published with the manuscript if accepted.**

Author response: Confirming this has been completed.

***       Please note that PLOS Medicine requires prospective, public registration of a data sharing plan (as part of mandatory clinical trials registration) for all clinical trials that began enrollment on or after January 1, 2019, in accordance with ICMJE requirements.**

Author response: Confirming this has been completed.
