# Supplementary material for: Effects of X-ray–based diagnosis and explanation of knee osteoarthritis on patient beliefs about osteoarthritis management: A randomised clinical trial
Source: PLoS Med. 2025 Feb 4;22(2):e1004537. doi: 10.1371/journal.pmed.1004537 (PMC11838874; doi:10.1371/journal.pmed.1004537)
Supplement: Attachment — Submitted filename: PLOS Medicine response to reviewer comments_v2.docx [file pmed.1004537.s016.docx]

16^th^ January 2024

Dear Dr Van Epps,

Re: PMEDICINE-D-24-03202R1 Manuscript revisions

We are very pleased to hear that our manuscript is provisionally accepted for publication in *PLOS Medicine*, and we thank the editors and reviewers for their work improving the quality of our manuscript*.* In the following pages, we have outlined our responses to the editor requests.

Yours sincerely,

Belinda Lawford

On behalf of the authors

**Requests from Editors:**

**1.      Abstract, line 45 (and author summary). Please consider replacing ‘considered’ with ‘were presented with’ (ie, “Participants were presented with a hypothetical scenario…”).**

Author action: We have replaced ‘considered’ with ‘were presented with’ in the abstract and author summary as suggested.

**2.      Abstract line 53-54. If you wish to include the secondary comparison (clinical explanation [no x-rays] versus radiographic explanation [not showing x-ray images]) in the methodology paragraph, you should report the corresponding outcomes. Alternatively, you can remove the secondary comparison from the abstract (per CONSORT).**

Author action: We have removed this secondary comparison from the abstract.

**3.      Please add a sentence at the end of the Abstract/Methods and findings section indicating the main limitation(s) of the trial.**

Author action: We have added a sentence to the end of the methods and findings section of the abstract indicating the main limitations as suggested:

“*Limitations of our study included the fact that participants were responding to a hypothetical scenario, and so findings may not necessarily translate to real-world clinical situations, and that it is unclear whether effects would impact subsequent OA management behaviours*.”

**4.      Please review your text for claims of novelty or primacy (e.g. 'for the first time') and either remove this language or include “to our knowledge”—for example in the author summary and Discussion, line 390. In addition, please check that any use of statistical terms (such as trend or significant) are supported by the data, and if not please remove them.**

Author action: This language has been edited as suggested (to include the words ‘to our knowledge’). We have also checked and not found any statistical terms that are not supported by the data.

**5.      Please remove subheadings from the Discussion section, which should be presented as a continuous narrative.**

Author action: These have now been removed as suggested.

**6.      Please include URLs for relevant references (eg, refs 5, 6, 18, etc).**

Author action: These have now been added as requested.

**7.      Please ensure that the link to the Dryad Digital Repository is live when you submit the final revision.**
Author action: Please note that we have now uploaded our data on figshare, rather than the Dryad Digital Repository as we had not realised Dryad was not a free service. We have updated the manuscript to reflect this, and the link is live.

**8.      Please modify the CONSORT checklist to use section and paragraph numbers, rather than page numbers.**

Author action: This has been modified as requested.

**9.      Please upload the supplemental material as a separate file to the main manuscript.**

Author action: This has now been uploaded as a separate file as requested.

**10.     In accordance with ICMJE requirements, PLOS Medicine requires prospective, public registration of a data sharing plan (as part of mandatory clinical trials registration) for all clinical trials that began enrollment on or after January 1, 2019.**

Author action: Our clinical trials registry record (ACTRN12624000622505) has information about data sharing. We have updated this record to include a link to the data on the repository. Please note that updates to our registry have to be approved by ANZCTR, and may take up to 4 weeks to be reviewed (and therefore may not yet be live on the registry website). Please let us know if that is not what is meant by this comment.
